# Reading Akkadian cuneiform using natural language processing

**DOI:** 10.1371/journal.pone.0240511

**Published:** 2020-10-28

**Authors:** Shai Gordin, Gai Gutherz, Ariel Elazary, Avital Romach, Enrique Jiménez, Jonathan Berant, Yoram Cohen

**Affiliations:** 1 Faculty of Social Sciences and Humanities, Digital Humanities Ariel Lab, Ariel University, Ariel, Israel; 2 School of Computer Sciences, Tel Aviv University, Tel Aviv, Israel; 3 Jacob M. Alkow Department of Archaeology and Ancient Near Eastern Civilizations, Tel Aviv University, Tel Aviv, Israel; 4 Institute for Assyriology and Hittitology, Ludwig-Maximilians-Universität München, Munich, Germany; University of Modena and Reggio Emilia, ITALY

## Abstract

In this paper we present a new method for automatic transliteration and segmentation of Unicode cuneiform glyphs using Natural Language Processing (NLP) techniques. Cuneiform is one of the earliest known writing system in the world, which documents millennia of human civilizations in the ancient Near East. Hundreds of thousands of cuneiform texts were found in the nineteenth and twentieth centuries CE, most of which are written in Akkadian. However, there are still tens of thousands of texts to be published. We use models based on machine learning algorithms such as recurrent neural networks (RNN) with an accuracy reaching up to 97% for automatically transliterating and segmenting standard Unicode cuneiform glyphs into words. Therefore, our method and results form a major step towards creating a human-machine interface for creating digitized editions. Our code, Akkademia, is made publicly available for use via a web application, a python package, and a github repository.

## Introduction

**Fig 1:**
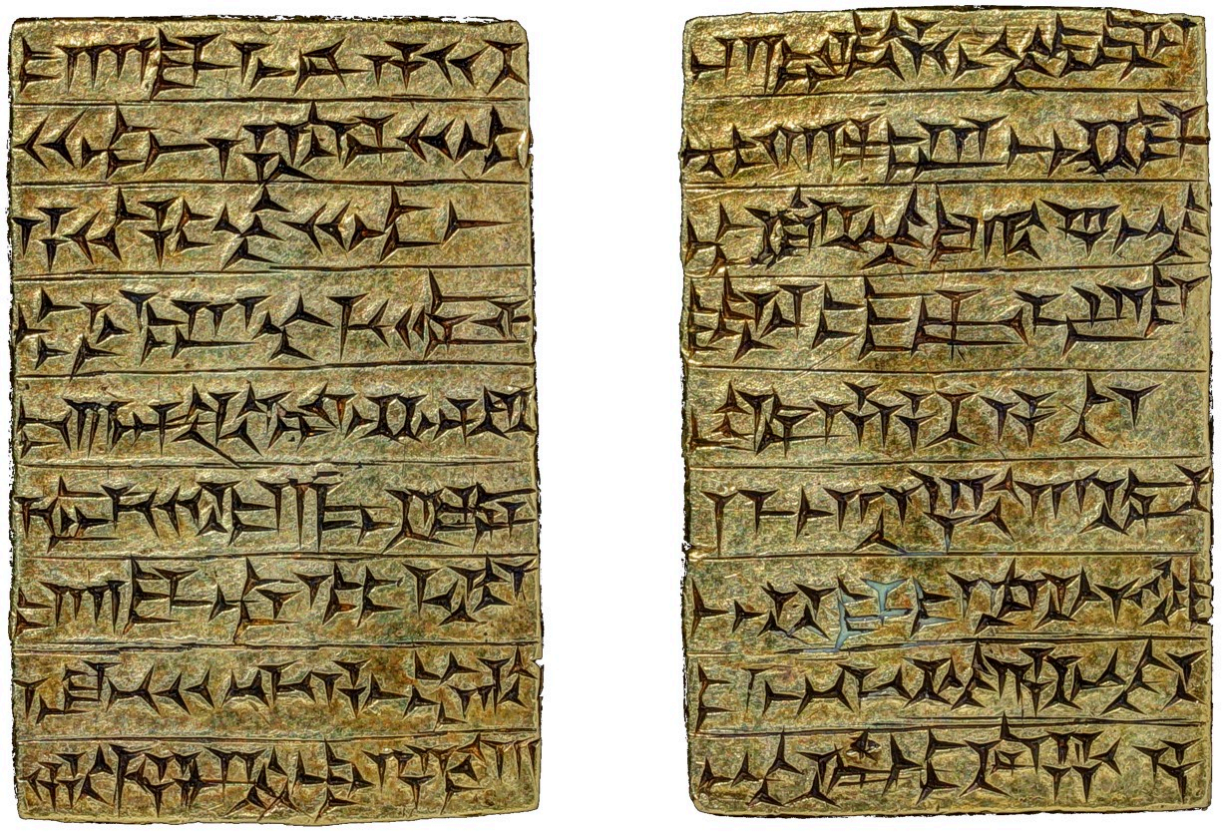
Example of cuneiform writing from a gold tablet of Ashurnaṣirpal II. This is a foundation inscription buried under the palace of Ashurnaṣirpal in the city Apqu (modern Tell Abu Marya, see link [3]), where it was found millennia later by archaeologists. After giving the titles of the king, the text introduces itself as a foundation inscription, written on gold and silver (the silver parallel of this copy was found alongside the gold). This is an anomaly for cuneiform texts, which were usually written on clay. Therefore, these inscriptions are a symbol of great wealth and extravagance. It ends with curse formulae for later princes who may dare to erase Ashurnaṣirpal’s name from his inscriptions. It is currently in the Yale Babylonian collection (YBC 2398; YPM BC 16991), republished from the Yale Peabody Museum of Natural History under a CC BY license, with permission from Klaus Wagensonner, original copyright 2020.

Cuneiform is one of the earliest forms of writing known in mankind’s history. It was used to write one of the main languages of the ancient world, Akkadian, which belongs, like Hebrew and Arabic, to the Semitic language family. About 2,500 years of human activity have been recorded in several dialects of this language across most of the ancient Near East. In all, at least 600,000 inscribed clay tablets and hundreds of inscriptions on stones and other materials are held in museums around the world. Akkadian is our main cultural source of the most prominent civilizations of the ancient Near East: these include the Akkadian Empire of Sargon in the third millennium BCE, the Empires of the Late Bronze Age for which Akkadian served as *lingua franca*, and the Neo-Assyrian, Neo-Babylonian and in part also the Persian Empires. A wide variety of texts have been written in Akkadian, such as royal inscriptions, literature, scientific and lexical compendia, wisdom compositions and liturgical literature, correspondence, and legal, administrative and economic documents. From each of these genres different aspects of life in ancient Mesopotamia may be gleaned. Even during the Hellenistic period, textual production continued, mostly in Babylonian temple communities: the last dated cuneiform documents were written during the first century CE [1, 2]. Thus, cuneiform sources are relevant to the early history of mankind and to the history of the ancient Middle East in its cultural context. A photograph of a cuneiform tablet for the sake of illustration can be seen in Fig 1.

In spite of their importance, many cuneiform documents remain unpublished, and the troves of information they hold are limited to well-studied corpora and historical periods. This is partially because the state of preservation of the materials is sometimes poor, making it difficult to read the signs or characters impressed on basically a 3D surface. This renders the reading process difficult and cumbersome. Furthermore, there are additional difficulties regarding the nature of the cuneiform writing system, see section Problem Statement.

In this article, we present the results of our joint project for automatically transliterating and segmenting Unicode cuneiform glyphs (for the creation of Unicode cuneiform, range U+12000—U+123FF, see [4]). The end goal in mind is to create a tool which can assist scholars making text editions. This tool will offer the most likely transliteration which will need a minimal amount of corrections. Therefore, it will quicken the process of publishing texts and making them available for scholarship.

### Problem statement

The translation of cuneiform texts into modern languages requires three steps: (1) a sign-by-sign transliteration; (2) word segmentation, i.e. joining the individual sign readings into words which are expressed by a hyphen (-) or a dot (.); (3) translation into a modern language. The first stage is vital because of the polyvalent nature of the cuneiform script, i.e. almost every sign has different possible readings.

Generally speaking, cuneiform sign-values can be divided into three categories: (1) logographic, one sign (or a combination of signs) representing one word, and usually written in capital letters in modern transliterations; (2) phonetic, one sign representing one syllable, and usually written in italics in transliterations; and (3) determinatives, which are semantic classifiers whose purpose is to aid the correct reading of the word which either follows or precedes them. In transliterations, they are written in superscript. Thus, while modern scholarly convention differentiates between logograms, phonetic readings, and determinatives, the cuneiform signs are exactly the same for all three. For example, the sign 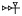 can be read as the logogram DINGIR, meaning “god”; it can be read syllabically *an*, as part of a word; and it can be a determinative ^d^, appearing before divine names. For example, the name of Sîn, the Babylonian moon god, will be written 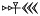 and it will appear in transliteration as ^d^30 (the moon god was written logographically with the sign for the number 30; the number of days in a lunar month).

Additionally, each of these categories can contain more than one value for any given period or genre of the Akkadian language. Thus, the same sign 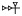 can be read logographically DINGIR, “god”, but also AN, “sky”. In addition to the most common phonetic reading *an*, which is found in texts from all periods, the sign has nine more phonetic readings, some of which are only found in particular periods, regions or genres (for example, *ilu* in Nuzi, *le*_4_ in El-Amarna, or *šubul* in scholarly texts).

The process described above, of determining appropriate sign readings by looking at the context from previous and following signs, can be efficiently solved by NLP tagging algorithms, as our results show. Therefore, the results of our project can make a significant step toward rediscovering the lost heritage of the ancient Near East. Our models receive as input a string of standard cuneiform Unicode glyphs and produce a segmented transliteration. In order to train the models, we used significant amounts of existing transliterations for which strings of cuneiform Unicode glyphs have already been generated, see Related Work and Materials and methods. We release all code and data used during this project, see here.

### Related work

Automating the process of making cuneiform text editions requires a pipeline which deals with each of the following three problems with different AI models: (1) extraction and visual recognition of 2-dimensional or 3-dimensional representations of cuneiform signs; (2) automatic transliteration and segmentation; (3) translation and/or annotation. In our project, we offer a solution for the second problem. For each of these steps, machine learning models require large amounts of training data. The following section presents a brief overview of the current availability of digitized cuneiform data and the state-of-the-art on each stage in the pipeline.

The largest repository of cuneiform digital textual corpora is the Open Richly Annotated Cuneiform Corpus (ORACC). It contains tens of thousands of texts with varying levels of transliteration, translation, annotation, and metadata in at least 20 different scholarly projects. However, one should bear in mind that ORACC is far from comprehensive for the entire cuneiform corpus. The Neo-Assyrian period is the most prevalent on ORACC; made recently available on a wide variety of formats (TEI/XML, JSON and TXT) thanks to the Munich Open-access Cuneiform Corpus Initiative (MOCCI). The Cuneiform Digital Library Initiative (CDLI) offers more than 300,000 text entries on their website, mostly with metadata only. Many entries contain low-resolution pictures of the tablets or only their hand copies (black and white drawings depicting signs in 2D). Less then half of the entries include transliterations and some include translations. There are many more online resources and databases for cuneiform studies, too many to mention in the scope of this article. For a comprehensive survey up till 2014, see D. Charpin [5].

The first stage of the pipeline for automatically creating cuneiform text editions requires procuring digital representations of cuneiform tablets. Currently, these are 3D scans, 2D or 2D+ photographs, and images of hand-copies: drawings made by Assyriologists which imitate the tablet in a 2-dimensional, black and white presentation. Hand-copies are still a standard in the publishing of traditional cuneiform text editions, and therefore they exist in abundance. They were the most cost-effective method for publishing a visual representation of cuneiform tablets until recent years. The situation, however, is very different for 3D and 2D representations. Several major projects developed effective methods for 3D or 2D+ scans and photographs of cuneiform tablets, such as the pioneering, but now defunct iClay project of J. Cohen *et al*. [4]. The leading methods for acquiring 2D+ images are currently the Polynomial Texture Mapping (PTM) and Reflection Transformation Imaging (RTI) dome shaped systems developed independently by G. Earl *et al*. [6], on the one hand, and by H. Hameeuw and G. Willems [7], on the other. Furthermore, there are two leading research groups creating 3-dimensional scans of tablets, that are developing methods of sign extraction and identification. A joint Dortmund-Würzburg team, led by G. Müller, G. Fink and F. Weichert, made 3-dimensional scans of cuneiform fragments and created the CuneiformAnalyser. It displays 3-dimensional scans of tablets, enhances features, extracts and recognizes signs and also reconstructs fragmentary tablets [8–10]. Another group from Heidelberg, led by H. Mara, developed the GigaMesh software, which also displays 3D scans of cuneiform tablets and provides philological tools such as sign extraction [11, 12]. In addition, they are developing methods for sign identification, both from 2D projections of 3D scans and hand-copies [13–17]. The best results are usually attained with 3D scans, but there are not enough scanned tablets. 3D scans are still expensive to produce in terms of the equipment necessary, and are also time-consuming, since the high-quality required for machine learning algorithms is considerable and this lengthens the duration of individual scans. However, there have been developments in the field of photogrammetry: there are now cheaper and quicker methods for creating 3D models, which do not require a specialized scanner, but rather use a turntable and standard cameras to create a 3-dimensional scan from multiple pictures, sometimes achieving a higher quality image than a standard 3D scanner, see T. Collins *et al*. [18].

After signs are extracted and identified, they need to be transliterated and segmented. The first research team to attempt transliteration of cuneiform texts are B. Bogacz *et al*. [19]. They attempted a pipeline of sign extraction, identification, and transliteration: they took 30 raster images of hand-copies with their corresponding transliterations from the Cuneiform Commentary Project (CCP) website, visually segmented the signs using Histogram of oriented Gradients (HoG) feature descriptors, and labeled them to their corresponding transliteration for training. Their best-performing algorithm was a Hidden Markov Model (HMM), but nevertheless, they achieved low accuracy scores.

The first to attempt segmentation are T. Homburg and C. Chiarcos [20]. They used rule-based, dictionary-based, and machine learning algorithms in order to segment the input of Unicode cuneiform glyphs, prepared from texts available at the CDLI website. They took texts from the Old Babylonian, Middle Babylonian and Neo Assyrian periods, and trained and tested each time-period individually. Their results for texts of different periods varied, but overall the best performing algorithms were dictionary-based algorithms, originally developed for Chinese and Japanese, with an F-score averaging between 60%-80%. They also attempted transliteration, by developing a baseline formed on the most common transliteration for each sign that reaches an accuracy of 40%. Therefore, in this paper we present our state-of-the art approach for automatically transliterating and segmenting Unicode cuneiform glyphs.

The only other project using Unicode cuneiform characters that we are aware of is T. Jauhiainen *et al*. [21], a task force for language identification in cuneiform texts which was part of the VarDial 2019 workshop. The Project used as data sets texts from ORACC in the Sumerian language and in six dialects of Akkadian. Out of the groups which participated in the task force, the third, fourth, and fifth places published their results in [22–24], respectively.

To our knowledge, there have been no publications attempting automatic translation of texts written in cuneiform. There have been various studies on the automatic annotation of cuneiform texts written in the Sumerian language, e.g. [25–28]. Currently, a research team is working on the project Machine Translation and Automated Analysis of Cuneiform Languages (MTAAC), led by H. Baker, C. Chiarcos, R. Englund, and É. Pagé-Perron: see [29, 30] and their website.

## Materials and methods

We have used for our project the Royal Inscriptions of the Neo-Assyrian Period (RINAP) from ORACC, based on data prepared by the Humboldt Foundation-funded Official Inscriptions of the Middle East in Antiquity (OIMEA) Munich Project of Karen Radner and Jamie Novotny, see link. These royal inscriptions cover the reigns of Tiglath-Pileser III (744-727 BCE), Shalmaneser V (726-722 BCE), Sennacherib (704-681 BCE), Esarhaddon (680-669 BCE), Ashurbanipal (668-ca. 631 BCE), Aššur-etel-ilāni (ca. 631–627/626 BC), and Sîn-šarra-iškun (627/626–612 BC).

These contain overall 23,526 lines, where we treat each line as an example. We divided each corpus in the following manner: 80% for training (18,822 lines), 10% for validation (2,352 lines) and 10% for testing (2,352 lines). For a full breakdown of the corpora, see Table 1. These corpora also have an encoded Unicode version of the cuneiform. This was generated by Cuneify, a tool created by Steve Tinney, which generates strings of Unicode cuneiform glyphs from Roman characters representing a transliteration. Thus, we used unsegmented strings of encoded Unicode cuneiform as our input, and the output is the transliteration with segmentation in Roman characters.

**Table 1:** Breakdown of RINAP corpora.

	RINAP 1	RINAP 3	RINAP 4	RINAP 5
**Training Percentage**	3.83%	24.25%	20.46%	31.46%
**Training Lines**	900	5,705	4,814	7,402
**Validation Percentage**	0.48%	3.03%	2.56%	3.93%
**Validation Lines**	112	713	602	925
**Test Percentage**	0.48%	3.03%	2.56%	3.93%
**Test Lines**	112	713	602	925
**Overall Percentage**	4.78%	30.31%	25.58%	39.33%
**Overall Lines**	1,125	7,131	6,018	9,252

**Fig 2:**
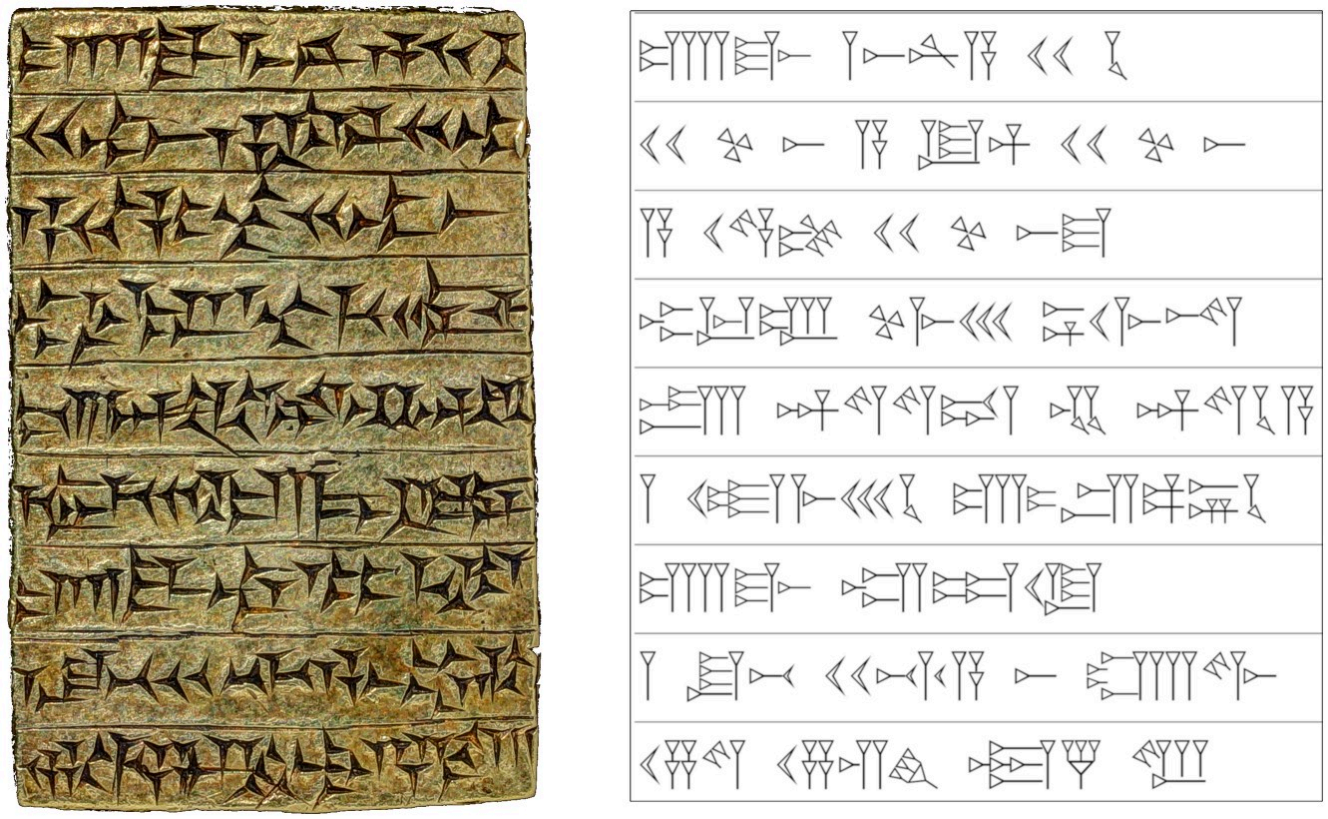
Example of Unicode cuneiform. On the left are the original cuneiform characters written on the obverse of the gold tablet introduced in Fig 1. On the right is the Unicode version which was generated from the transliteration of this inscription.

It is important to note that the cuneiform “signs” used in this study are a digitized Unicode representation of sign values as opposed to sign forms (see Fig 2). The visual identification of the cuneiform syllabary, which will enable identifying signs on original tablets with their Unicode equivalent remains outside the purview of the current study, and would require a separate model (see review of previous literature in section Related Work).

We did not use an external dictionary which contains all possible values for each sign. Instead, we built a dictionary from the data itself: each sign was mapped to the set of transliterations that co-occurred with it in the training set. We restricted it so that each sign can only have those possible transliterations, rather than having all the transliterations in the corpus as a possible value for each sign. We assume that all transliterations of the test set appear in the training set as well; a reasonable assumption to make.

Restricting to specific transliteration is also important as the number of overall sign-values is high (almost 3,000). Thus computing bigram and trigram statistics for all transliterations is computationally prohibitive (a bigram is a sequence of two adjacent transliterations and a trigram is a sequence of three). It is also unnecessary, since each sign has a unique set of possible transliterations, none of which are shared with any other sign. Restricting the number of possible transliterations for each sign dramatically improves computation efficiency.

We took into consideration word separators, i.e. whether a sign has a hyphen or a dot after it, so that we can also determine segmentation (see below under Formulation as a Tagging Problem). The transliterations also include signs which are not visible on the clay tablet, but are reconstructed by the modern editor. These are marked in a particular way: broken signs are inside square brackets and partially missing signs are inside half brackets. During our research we did not take into consideration these brackets, but used all the transliterated signs as the right and complete option.

### Formulation as a tagging problem

We assume each Unicode glyph corresponds to one sign in the transliteration, in order to cast the problem as a tagging problem, where each sign is marked, or *tagged*, by its proper transliteration. However, the cuneiform writing system can use compound signs, creating new meaning with the combination of two or more signs, much like the Chinese writing system. For example, the logogram SIPA (“herdsman”) is made of two signs, 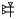 PA and 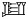 LU, and the logogram ENSI_2_ (“governor”) is made of three, 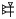 PA, 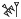 TE and 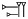 SI. In Unicode cuneiform, as in actual cuneiform, the compound signs are not shown as one character, but rather they are formed from a group of characters. As a result, these compound signs are read together as one sign in the transliterations, which results in an alignment problem.

Thanks to the efforts of the OIMEA project team at LMU Munich, the RINAP texts are also available in JSON format on their website. In the JSON files we could find each sign or group of signs with their matching transliteration. This resolved the alignment problem. To handle these compound signs, we used the BIO-tagging scheme [31], common in Named Entity Recognition (NER) tagging in NLP, which allows tagging a sequence of contiguous signs by the same tag. We used an index for each such sign reading to mark they are all part of the same transliteration. For example, the two signs 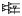 (DUMU, “son”) and 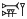 (GEŠ_3_, “male member”) that should be read together as the logogram IBILA (“descendent”) appear as IBILA(0) and IBILA(1). In the output, only IBILA(0) is shown, since there is no need for the transliteration to appear twice.

To handle both transliteration and word segmentation in a single model, we double the number of possible transliteration symbols, where for every transliteration *t* we add a symbol $\hat{t}$ (representing a sign with a hyphen or a dot after it). This denotes that this tag is not the last tag of a word. Thus, after tagging a sentence, we can decode both the transliteration and segmentation.

### A Hidden Markov model

A Hidden Markov Model (HMM) is a classical statistical model [32] that embodies a set of statistical assumptions concerning the generation of sample data (and similar data from a larger population). It is often used in NLP for tagging [33, 34]. It can be applied to speech recognition, part-of-speech tagging and various bioinformatics applications *inter alia*. Given an input sequence of *n* signs *X* = (*x*_1_, …, *x*_*n*_) and a possible output transliteration *Y* = (*y*_1_, …, *y*_*n*_), an HMM provides the probability *p*(*X*, *Y*). Thus, given a sequence of signs *X* = (*x*_1_, …, *x*_*n*_), we can choose the transliteration $\hat{Y}$ that maximizes $p(X,\hat{Y})$.

HMMs factorize the probability *p*(*X*, *Y*) into transition probabilities and emission probabilities. Concretely, we use a trigram HMM:

$$\begin{array}{c} P\left(X,Y\right)=\prod_{i=1}^{n}e\left(x_{i}|y_{i}\right)q\left(y_{i}|y_{i-1},y_{i-2}\right) \end{array}$$

where *e*(⋅) is the emission probability of observing the sign *x*_*i*_ given the transliteration *y*_*i*_, and the transition probability *q*(*y*_*i*_|*y*_*i*−1_, *y*_*i*−2_) is the probability of the transliteration *y*_*i*_ given the previous two transliteration. The probabilities *q*(⋅) and *e*(⋅) are estimated using maximum likelihood from a training corpus, and the predicted sequence $\hat{Y}$ that maximizes $P(X,\hat{Y})$ is found using the Viterbi algorithm (A dynamic programming algorithm for finding the most likely sequence according to a model, used as part of the HMM and MEMM algorithms [35]). Thus, an HMM captures the correlations between signs and transliterations, as well as which transliteration trigrams (triplets of adjacent transliterations, hence the name *trigram HMM*) are likely according to the data.

Choosing the order of an n-gram model (trigram in our case) depends on two factors: (a) *computational efficiency*: using a higher order model (for example, 4-grams) makes decoding slower. (b) *statistical efficiency*: given infinite data, higher order models are more accurate. However, with limited amount of data, using high-order models quickly leads to *sparsity*—many n-grams are never observed in the training data, leading to poor performance. Thus, we use in this work trigram models, which empirically lead to good performance and an efficient model.

Because the number of possible transliterations for every sign is large, there are cases where the probability *p*(*X*, *Y*) is zero for all transliteration sequences *Y* (for example, when for a pair of signs all possible transliteration bigrams have not been observed in the training set). In this case, we use a backoff mechanism, where we choose the most common transliteration for each sign.

### A Maximum-entropy Markov model

A Maximum-entropy Markov model is a graphical model for sequence labeling that combines features of hidden Markov models (HMMs) and maximum entropy (MaxEnt) models. It is a model that outperforms HMMs as it can capture richer information [36] [37]. In MEMM, we directly estimate a conditional distribution

$$\begin{array}{c} P\left(Y|X\right)=\prod_{i=1}^{n}p\left(y_{i}|y_{i-1},y_{u-2},X\right), \end{array}$$

where the probability *p*(*y*_*i*_|*y*_*i*−1_, *y*_*u*−2_, *X*) is estimated as

$$\begin{array}{c} p\left(y_{i}|y_{i-1},y_{u-2},X\right)\propto s\left(X,y_{i-2},y_{i-1},y_{i}\right), \end{array}$$

and the scoring function is linear: $s\left(X,y_{i-2},y_{i-1},y_{i}\right)=\sum_{j=1}^{F}w_{j}f_{j}\left(X,y_{i-2},y_{i-1},y_{i}\right)$. Each feature *f*_*j*_() is extracted from the input sequence and the transliteration trigram, and the parameters *w*_*j*_ are learned.

Because *f*_*j*_() is an arbitrary function of its inputs, richer features that are useful for prediction can be used. For example, a feature can capture the correlation between a sign and the transliteration of the following sign, which is difficult to do with HMMs. Learning is done by maximizing the L2-regularized maximum likelihood objective over the training set, and the best sequence is found again using Viterbi which finds $\hat{Y}$ that maximizes $P(\hat{Y}|X)$.

The main advantage in MEMMs is the ability to define arbitrary and flexible features [38]. In this work, we found the following features to be most useful for our model *p*(*y*_*i*_|*y*_*i*−1_, *y*_*u*−2_, *X*): the current sign, the last two signs, the next two signs, and the transliterations’ bigram and trigram. We also added some domain-specific features that are based on the error types we observed in the HMM algorithm:

- Is the previous sign the last sign of a word or does the word continue with the current sign?
- Is the transliteration of the previous sign phonetic or is it a logogram? There are many cases in which a transliteration may be both (see below under error analysis).
- Is the transliteration of the previous sign a determinative? This may change the context of the word.
- Is the transliteration of the previous sign a compound sign?

While we used Viterbi as our inference algorithm, we also experimented with a greedy inference algorithm that simply chooses the best transliteration for each sign from left-to-right. This inference algorithm does not guarantee theoretically that the output will maximize $P(\hat{Y}|X)$, but is much faster than Viterbi. In practice, we found its quality is almost as high as Viterbi.

### A neural sequence model

In recent years, deep learning models based on neural networks, which allow to capture complex dependencies between inputs and outputs, have improved performance and become standard in NLP. Tagging is no exception, where a common approach for modelling problems in which the input is a sequence, is to use a Recurrent Neural Network (RNN), using the Long-Short Term Memory (LSTM) cell [39]. The advantages of LSTM are: (a) they can capture long-range dependencies, while HMMs and MEMMs only look at a bounded window around every position; (b) the class of functions that they express is larger.

**Fig 3:**
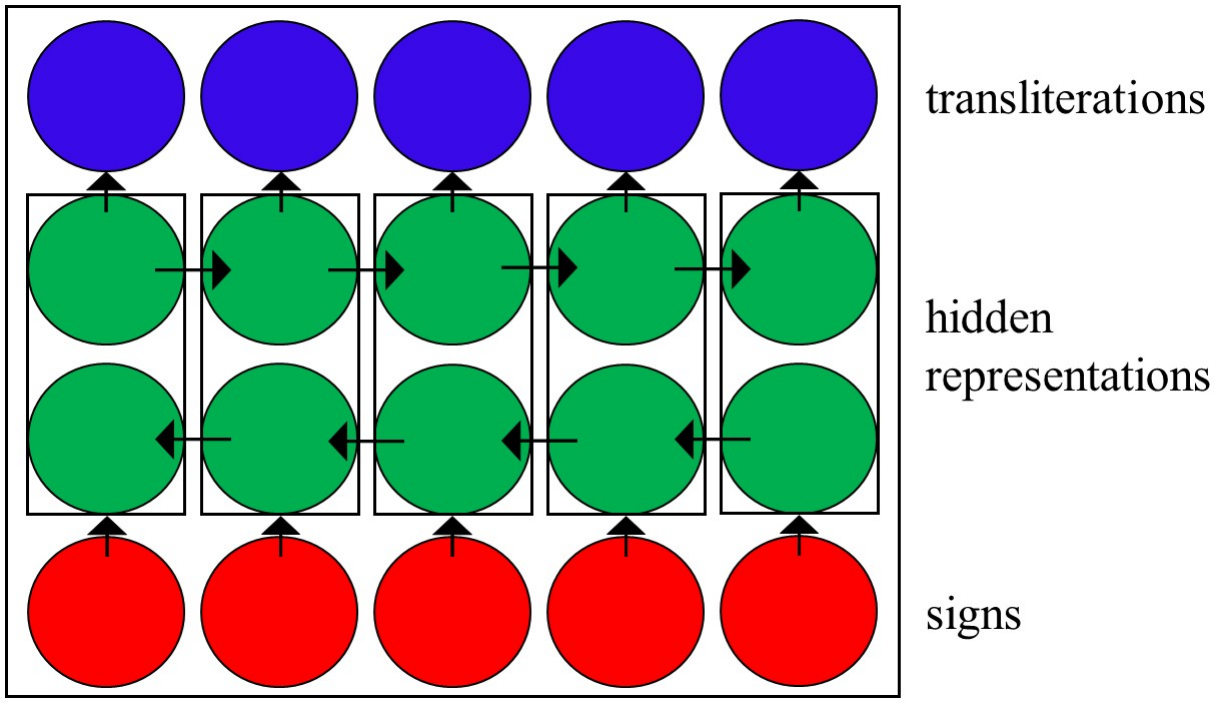
An illustration of the bidirectional LSTM.

In an LSTM, a neural network “reads” the input sequence word by word from left-to-right, and creates a representation for every word that is conditioned on all previous words. Thus, each word depends on the entire history and can capture long-range dependencies. We use a Bidirectional LSTM, that is, one LSTM reads the input from left to right, and another reads the input from right to left: see Fig 3. Thus, the representation of every word depends on all of the words that occur both before it and after it, which has been shown to be useful in many tasks, including tagging [40, 41]. Once these representations are created, we predict the transliteration in every position from the learned representations. Training is done again using maximum likelihood over the training data.

We adapt the BiLSTM from the open source deep learning library AllenNLP for our purposes [42]. We performed hyper-parameter search for the word embedding size, hidden representation size and learning rate, by doing grid search and choosing the values that maximize performance on a development set. We use 200-dimensional vectors as the word embedding size as well as the hidden representation size, and a learning rate of 0.3.

We optimized the learning rates, the embedding size, and hidden representation size. Details regarding how accuracy changes for different parameters can be found in the S1 File. We observe that a smaller dimension size dramatically reduces performance, while a larger dimension size only slightly reduces performance, but leads to a longer training time. The learning rate of 0.3 gave best results compared to other options on the development set.

## Results and discussion

Table 2 shows the results of our models. Results for individual datasets are available in the S1 File. The first row reports accuracy when both transliteration and segmentation are taken into account. It is computed by the sum of correct sign taggings divided by the total number of signs. In the second row we only take into account the correct transliteration, ignoring whether or not the segmentation is correct (i.e. whether it is (*t*) or ($\hat{t}$)). As before, it is computed by the sum of correct sign taggings divided by the total number of signs except for one difference—correct tagging is agnostic to segmentation. In the last row we report results on segmentation only using the F_1_ measure, which is more natural for segmentation—the harmonic mean between precision and recall in our model.

**Table 2:** Test accuracy for all models.

	HMM	MEMM	BiLSTM
**transliteration and segmentation (accuracy)**	89.5%	94%	96.7%
**transliteration only (accuracy)**	93.6%	96.4%	97.8%
**segmentation only (F_1_)**	91.8%	95.9%	97.9%

Specifically, if we define *true positives* (tp) to be the number of signs that end words that are identified correctly, *true negatives* (fn) to be the number of signs that end words that are identified incorrectly, and *false positives* (fp) to be the number of signs that are incorrectly identified as a word end, then:

$$\begin{array}{c} \text{precision}=\frac{\text{tp}}{\text{tp}+\text{fp}},\text{recall}=\frac{\text{tp}}{\text{tp}+\text{fn}},F_{1}=\frac{2\cdot \text{precision}\cdot \text{recall}}{\text{precision}+\text{recall}}. \end{array}$$

**Fig 4:**
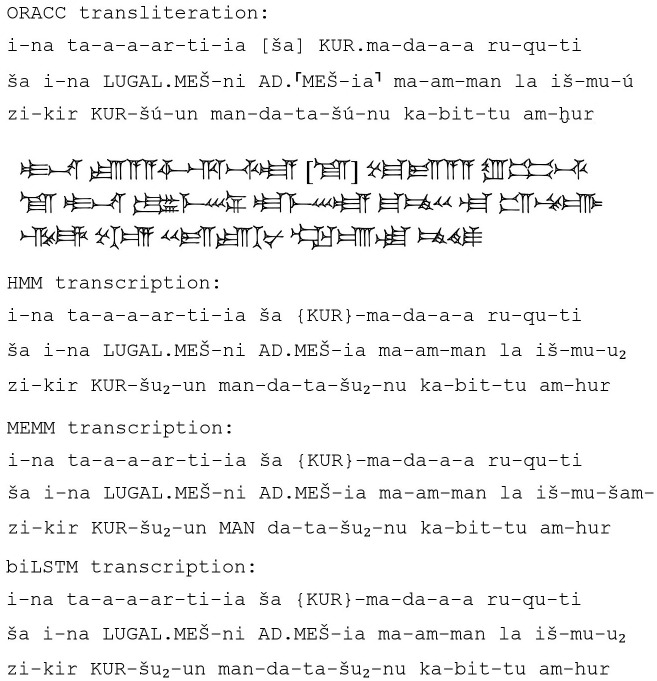
Output of the algorithms. These are lines 31-34 of the second column of Sennacherib’s clay prism, probably from Nineveh, now in the Israel Museum (IMJ 71.072.0249). The text records eight campaigns of the Assyrian King, including the siege of Jerusalem which is well known from the Book of Kings. The line reads: ‘On my return march, I received a heavy tribute from the distant Medes, of whose land none of the kings, my ancestors, had heard mention.’ (translation adapted from A.K. Grayson and J. Novotny’s edition available on ORACC, Q003497).

As expected, the best results are obtained using the BiLSTM model, then the MEMM, and last, but still quite impressive results, the HMM. An example of our algorithms’ output is shown in Fig 4.

### Error analysis

We recognize four main types of errors:

- The most common error is word segmentation, as there is an almost 4% difference between total accuracy and transliteration only accuracy in HMM for example. The algorithms tend to transliterate the signs well but they sometimes do not segment the words properly. The main reason for that can be that these words do not have a typical suffix such as a case marker or number. Many examples of different contexts are needed in order to improve our results.
- Some signs can have the same value in their logographic and phonetic reading. For example, 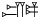 has the logographic reading SAG (‘head’), and also the phonetic reading *sag*. The algorithms can correctly identify “sag”, but cannot identify correctly whether it should be a logogram or a phonetic reading.
- The same type of error occurs with determinatives. For example, 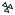 can be a logogram KUR (‘country’), or the determinative ^kur^ which appears before names of lands (and some cities).
- errors appear more often with compound signs. This is not surprising, considering that HMM takes into consideration the two previous signs and MEMM takes into consideration the two previous signs and the two following signs: if there is a complex sign made from two or three signs, it reduces the observable context.

It is important to note, however, that some of these apparent errors are to be evaluated on a case-by-case basis. We need to remember that the choice between reading a sign as a logogram or a determinative is a matter of scholarly convention based on lexical and contextual analysis. For example, some scholars treat KUR as a logogram, transliterating: KUR *Hat-ti* and some treat it as a determinative: ^kur^ *Hat-ti*. The meaning is not changed (“the land of Hatti” or simply “Hatti”).

The same can also apply to choosing between a logogram or a phonetic reading, particularly in proper names. For example, the name of the temple of Marduk in Babylon, Esagila, may be transliterated E_2_.SAG.IL_2_ or *e*_2_ *-sag-il*_2_.

### Four additional case studies

After evaluating the successful results of the algorithms, we selected four case studies from later and earlier periods. Our purpose was to examine the accuracy of the algorithms on texts from corpora the models had not observed. Therefore, we wanted to examine if our models can be used on unfamiliar texts in order to speed up the process of mass transliterations. The principles of cuneiform writing, as elaborated upon, remained the same throughout its usage, but there are naturally variants in common signs and common sign readings, spelling, vocabulary, and formulae used.

We selected the four following texts from two sub-projects in the OIMEA project on ORACC, The Royal Inscriptions of Assyria online (RIAo) and The Royal Inscriptions of Babylonia online (RIBo). We chose four royal inscriptions from different periods, two of which are written in a different dialect and two are written with Babylonian orthography (as opposed to the Assyrian one of our training corpus):

- A Middle-Assyrian Royal Inscription of Aššur-rem-nišešu I, ca. 1398-1391 BCE, found in Assur (link).
- A Middle-Babylonian *kudurru* (boundary stone) Royal Inscription of Nebuchadnezzar I, ca. 1125-1104 BCE, found in Sippar (link).
- A Neo-Babylonian cylinder Royal Inscription of Nabonidus, ca. 551-542 BCE, found in Sippar (link).
- A Hellenistic Royal Inscription of Antiochus I, ca. 281-261 BCE, found in Borsippa (link).

The Results of the models are summarised in Table 3. They are very satisfactory, considering the differences between the case studies and the corpora used for learning. The complete output of the algorithms for these texts is published alongside our article, following a full qualitative evaluation, in S2 File. In what follows we present a summary of said qualitative evaluation.

**Table 3:** Accuracy on case-studies.

	HMM	MEMM	BiLSTM
**Middle-Assyrian (Q005708)**	58%	60%	68%
**Middle-Babylonian (Q006251)**	59%	58%	72%
**Neo-Babylonian (Q005422)**	61%	62%	77%
**Hellenistic (Q004179)**	70%	67%	84%

We have noticed, unsurprisingly, that the errors referred to in section Error Analysis are the most common. For example, segmentation errors are around 12%-17% in HMM, 11%-16% in MEMM, and 4%-9% in BiLSTM. Errors which need to be evaluated on a case-by-case basis (i.e. logogram/determinative or logogram/phonetic) are around 1%-3%. Additionally, some errors are predictable and can be manually corrected. One such example is sign readings the algorithms had not seen before: in Nabonidus’ Inscription ii l. 13, the first sign of the word *kallatu* (“bride”), 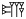, is unknown to the algorithm with the reading *kal*. HMM offers the reading *reb*, MEMM offers the reading KAL, and BiLSTM the reading *a*. As can be seen with this example, the models have two fall-back options: HMM and MEMM will use another reading of the sign from the dictionary. BiLSTM, on the other hand, has a “wild card” aspect, where it may offer a sign-reading based on the context and not on the sign-values in the dictionary, since it is not limited to the dictionary. In future, we will flag these readings and give the user the option whether they prefer to be given the best contextual reading or the best contextual reading when limiting the options to within the dictionary.

Furthermore, we have noticed that even when the algorithms output errors, the errors stay localized and they do not create a chain-reaction of errors. Were it otherwise, the algorithms would be of no use for corpora they have not learned. This should not be taken for granted, considering the algorithms are context-based.

To conclude, our models will not replace the scholar’s work. Rather, they will provide considerable assistance in speeding up the scholar’s work on transliteration and segmentation. Since our models provide 70% or more correct readings on unlearned corpora and 97% correct readings on learned corpora, minor errors like those expanded upon in S2 File can be easily corrected, and one can dedicate their time to lines which are more difficult to decipher (both for the algorithms and for scholars).

## Conclusion

We have succeeded to transcribe with very high accuracy (almost 97%) Unicode cuneiform characters to the appropriate segmented transliteration; this achievement comprises the first two stages of translating a text written in Akkadian cuneiform texts. Our results are state-of-the-art for machine transliteration and segmentation of cuneiform characters.

Our code, called Akkademia, is publicly available and can be deployed in three different ways: (1) via an online application at the Babylonian Engine website; (2) via a python package, called akkadian; (3) as a downloadable github repository. The latter includes the full code used to train the models and the possibility to train it on additional databases. Full explanation on how to download and employ the python package and github repository are available at the github project’s webpage.

Our next step forward is to train the models on larger corpora that are available in a suitable digitized format, or can be converted to such. Additionally, we plan to expand and improve the online application to make it as easy to use as possible, and have the user receive not just a transliteration of their input, but also a downloadable version of the transliteration in a digitized format (TEI/XML or JSON). We also plan to attempt employing our models for other functions, such as offering likelihood estimates for sign restorations. Currently, we are limited to the manual identification of signs from the original tablets. The user will have to look at the tablet or its representation, identify the signs and input them into the application. We hope to develop visual recognition algorithms that will assist this process in the future, and become part of a pipeline from tablet to digital edition which a scholar can work alongside of to assist and quicken the publication process.

There is a growing new approach where digital editions are a must in order to follow new avenues of research in the age of Big Data: text mining, social and geographical network analysis, and treebanks to name just a few. In other words, we wish to create an environment where human and machine can cooperate on scholarly work. Not just in the field of cuneiform studies is this shift vital, but also in the Humanities at large. Data has to be curated in a platform agnostic format for inner- and multidisciplinary applications. This is one step towards large scale experimentation with limited textual data. In the near future we will be attempting to expand our models across corpora and even across large spans of time, centuries and millennia.

## Supporting information

S1 File Additional results. Includes optimization of meta-parameters for BiLSTM and performance on individual datasets.
(PDF)

S2 File Four additional case studies. Our qualitative analysis of four texts from outside the training corpora with full output of the models.
(PDF)

S1 Data (PDF)
