# Supplementary material for: Reading Akkadian cuneiform using natural language processing
Source: PLoS One. 2020 Oct 28;15(10):e0240511. doi: 10.1371/journal.pone.0240511 (PMC7592802; doi:10.1371/journal.pone.0240511)
Supplement: S1 File — Includes optimization of meta-parameters for BiLSTM and performance on individual datasets. (PDF) [file pone.0240511.s001.pdf]

S1 File

Optimization of meta-parameters for BiLSTM

Accuracy of different learning rates:

	0.1	0.3	0.8
transliteration and segmentation (accuracy)	95.5%	**96.7%**	90.0%
transliteration only (accuracy)	97.1%	**97.8%**	93.7%
segmentation only (F1)	97.0%	**97.9%**	93.3%

Accuracy of different embedding dimension and hidden dimension:

	5	200	500
transliteration and segmentation (accuracy)	69.4%	**96.7%**	95.2%
transliteration only (accuracy)	79.2%	**97.8%**	96.5%
segmentation only (F1)	76.7%	**97.9%**	97.2%

Performance on individual datasets

The best results are obtained using the MEMM model, then HMM and BiLSTM. The main reason for that is that BiLSTM neural network is usually more impressive on big datasets. As expected, all three models' accuracies are correlated with the size of the dataset.

Transliteration and segmentation (accuracy):

	HMM	MEMM	BiLSTM
RINAP 1	90.2%	91.4%	87.7%
RINAP 3	97.4%	97.6%	96.5%
RINAP 4	92.2%	92.6%	90.9%
RINAP 5	96.1%	96.1%	95.5%

Transliteration only (accuracy):

	HMM	MEMM	BiLSTM
RINAP 1	94.1%	94.8%	91.1%
RINAP 3	98.6%	98.6%	97.6%
RINAP 4	95.1%	95.4%	93.7%
RINAP 5	97.7%	97.6%	96.9%

Segmentation only (F1):

	HMM	MEMM	BiLSTM
RINAP 1	91.5%	92.9%	91.7%
RINAP 3	97.8%	98.1%	97.4%
RINAP 4	94.1%	94.5%	94.2%
RINAP 5	97%	97.2%	97.2%
