# Supplementary material for: Reading Akkadian cuneiform using natural language processing
Source: PLoS One. 2020 Oct 28;15(10):e0240511. doi: 10.1371/journal.pone.0240511 (PMC7592802; doi:10.1371/journal.pone.0240511)
Supplement: S2 File — Our qualitative analysis of four texts from outside the training corpora with full output of the models. (PDF) [file pone.0240511.s002.pdf]

S2 File

Introduction

Satisfactory training on every genre and time period of texts is not necessarily possible in the near future, considering the myriad of cuneiform texts that do not have a digital edition and those which do not have an edition at all. Therefore, we examined how well our algorithms transliterate texts from unfamiliar corpora, testing whether they are nevertheless able to be of use. It is readily apparent that, although the algorithms cannot replace detailed scholarly readings and commentary on ancient texts, the advantage of using such a tool is to provide a good working transliteration which varies based on the training corpus.

In this supplementary file are adjoined the results of our algorithms on four texts outside of the corpora we learned (the Royal Inscriptions of the Neo Assyrian Period, hereafter RINAP). We begin with a general commentary about the advantages and disadvantages of the algorithms, based on their results with these texts. Following that, we bring the texts themselves, with the Unicode cuneiform glyphs that were used as input, the published transliteration which appears in ORACC, and the output of the three algorithms. The texts we have chosen are the following:

- A Middle-Assyrian Royal Inscription of Aššur-rem-nišešu I, ca. 1398-1391 BCE, found in Assur.[1]
- A Middle-Babylonian *kudurru* (boundary stone) Royal Inscription of Nebuchadnezzar I, ca. 1125-1104 BCE, found in Sippar.[2]

[1]http://oracc.org/riao/Q005708/html
[2]http://oracc.org/ribo/Q006251/html

- A Neo-Babylonian cylinder Royal Inscription of Nabonidus, ca. 551-542 BCE, found in Sippar.[3]

- A Hellenistic Royal Inscription of Antiochus I, ca. 281-261 BCE, found in Borsippa.[4]

These texts were chosen based on their spatial and chronological distinctness from RINAP: they are either earlier (Middle-Assyrian Royal Inscription of Aššur-rem-nišešu), later (Hellenistic Royal Inscription of Antiochus I), or written in a Babylonian orthography (Neo-Babylonian cylinder Royal Inscription of Nabonidus). The *kudurru* Royal Inscription of Nebuchadnezzar I is both an earlier text and written in a Babylonian orthography.

Results

The results of our algorithms are summarized in Supplementary Table 1

	HMM	MEMM	BiLSTM
Middle-Assyrian (Q005708)	58%	60%	68%
Middle-Babylonian (Q006251)	59%	58%	72%
Neo-Babylonian (Q005422)	61%	62%	77%
Hellenistic (Q004179)	70%	67%	84%

Supplementary Table 1: Accuracy on New Texts

We have taken a closer examination of the types of errors, and noticed certain patterns. First of all, the same common errors that appeared in the texts from RINAP appeared with these four texts, particularly differences between logographic/syllabic or logographic/determinative readings, and segmentation. Differences between logographic/syllabic or logographic/determinative readings account for about 1%-3% of errors, but often for the Assyriologist it makes no substantial difference.

Segmentation is the commonest error, being 12%-17% in HMM, 11%-16% in MEMM, and 4%-9% in BiLSTM. However, a person with a basic knowledge of Akkadian will often be able to immediately detect and correct these errors. For example, in Nabonidus' inscription i l. 18, none of the algorithms

[3]http://oracc.org/ribo/Q005422/html
[4]http://oracc.org/ribo/Q004179/html

correctly segmented the name of the legendary king Narām-Sîn, grandson of Sargon of Akkad who reigned over Mesopotamia during the late third millennium. This is obvious for scholars and thus quickly amended. Another such error is in ii l. 22 of the same inscription: HMM has identified most of the line as a single word (*lib*$_3$*-bi-šu-mah-šal-mi-iš-it-tal-lak-*), something which is highly unlikely. MEMM has separated between *lib*$_3$*-bi* and *šu*: a basic knowledge of Akkadian suffices to know that *šu* is not usually a word on its own and must go with *lib*$_3$*-bi*. Following the same logic, one will know to correct BiLSTM's reading, *lib*$_3$*-bi-šu*-MAH, since *lib*$_3$*-bi-šu* would be segmented from the logogram following it. Thus, although segmentation remains problematic, the initial transliteration gives sufficient groundwork to improve upon. It is also worthwhile to stress here that when viewing the output of the three algorithms at once, one can view the various options and choose the appropriate transliteration from the one and the best segmentation from the other.

There are additional errors that we are unable to quantify, but they should not necessarily be considered errors. For example, there are instances when more than one sign reading can be correct. In l. 10 of Antiochus' Inscription, the sign 𒈫 is transliterated in the published edition prepared by K. Stevens as the logogram MIN in ŠU-MIN, standing for the dual form of *qātu*, "hand". The algorithms, however, read the sign as '2' or 'II'. This makes no difference, of course. Similar confusion occurs often with numbers. Another error regards the matter of mimation. The algorithms may pick the value with mimation or the one without it. In Antiochus' Inscription l. 54, the algorithms read SIG$_5$-*tim*, and not SIG$_5$-*ti*$_3$. The difference between the two is based on the late date of the inscription, but would not be vital in the attempt of mass automatic transliteration.

Another type of error which is obvious to Assyriologists is vowel or consonant harmony. For example, in Nabonidus' Inscription ii l. 22, BiLSTM transliterated the verb *ed-tal-lak*. However, the sign transliterated *ed* also has the values *et* and *it*, and there is usually consonant harmony between consecutive signs. The same is true for vowels: in l. 14 of Aššur-rem-nišešu's Inscription, HMM and BiLSTM transliterate *e-ip-bu-šu-* and *e-ip-pu-šu* (respectively), although according to vowel harmony *e-ep-pu-šu* is expected.

Thus far we have discussed errors which we view as minor or even irrelevant. There are, however, passages that the algorithms particularly fail to transliterate correctly. Generally speaking, lines which have only phonetic readings are easiest for the algorithms. Lines which have combinations of

phonetic readings and logograms tend to throw the algorithms off, and also consecutive cvc signs (for example Nabonidus' Inscription ii l. 17).

Furthermore, there are sign readings the algorithms have not seen before. For example, in Nabonidus' Inscription ii l. 13, the first sign of the word *kallatu* ("bride"), 𒆗, is unknown to the algorithm with the reading *kal*. HMM offers the reading *reb*, MEMM offers the reading KAL, and BiLSTM the reading *a*. As can be seen with this example, the models have two fall-back options: HMM and MEMM will use another reading of the sign from the dictionary. BiLSTM, on the other hand, has a "wild card" aspect, where it may offer a sign-reading based on the context and not on the sign-values in the dictionary, since it is not limited to the dictionary. In future, we will flag these readings and give the user the option whether they prefer to be given the best contextual reading or the best contextual reading when limiting the options to within the dictionary.

A particular failure of the algorithms is their ability to transliterate correctly names that they have not seen before is quite poor. But the same is often true for the scholar, as personal names usually require more time to decipher. This is particularly relevant when they are foreign names (for example the spelling of Antiochus and his wife Stratonike, ll. 1 and 56, respectively). Moreover, there are other predictable errors when there is a sign or sign value the algorithms have not seen before. Obviously, they are unable to correctly identify those.

Lastly, the most problematic recurrent error we have detected is 'disappearing' signs. As we explained in the article under the Formulation of the Tagging Problem section, we have marked compound signs in a way which makes them not appear in the output if they are the second, third or so forth part of a compound sign. For example, the three signs which form the logogram ENSI_2, 𒉺 PA, 𒋼 TE and 𒋛 SI, would be transliterated $\mathrm{ENSI}_2(0)$, $\mathrm{ENSI}_2(1)$, and $\mathrm{ENSI}_2(2)$, respectively, and only $\mathrm{ENSI}_2(0)$ (the first sign, PA) will appear in the output as ENSI_2. Unfortunately, sometimes the algorithms erroneously identify a sign as the second, third or so forth part of a compound sign, even when the first sign of the compound sign does not appear before said sign. Therefore, the sign does not appear in the output at all, although one would expect it to be. This happens most often with signs which are also numbers (most notably ia_2). Although this may cause confusion when examining the output, for now we have left the programming of compound signs as it is, since it is nevertheless efficient for the cuneiform script.

Our plan in order to reduce the percentage of errors is to teach the al-

gorithms more texts from different time periods and genres. Since our algorithms are very efficient for learned corpora, this should dramatically improve its accuracy. There will always remain, however, the problem of texts that have been understudied and underpublished, and even in learned corpora there are slight errors. Therefore, the algorithms will never be able to replace the scholar's work completely, nor do we aspire to create such a scenario. We want our algorithms to be used in a human-machine collaboration: the algorithms offer a quick, efficient transliteration, on which the scholar can improve with the deeper linguistic level and commentary a machine is not likely to be able to achieve, at least not in the foreseeable future.

To conclude, we would like to emphasize that our accuracy for even unknown corpora mostly surpasses previous attempts of automatic segmentation and transliteration of the cuneiform script. Furthermore, the correct readings the algorithms are able to produce are sufficient to provide a groundwork for the scholar when he is dealing with a previously unread text. Therefore, we do not aim that the algorithms will replace the scholar. Rather, the algorithms will be able to assist and quicken the process of not only making editions, but also making those editions available online in a digitized form that will enable further research using digital humanities' tools.

A Middle-Assyrian Royal Inscription of Aššur-rem-nišešu I, ca. 1398-1391, found in Assur (Q005708)

l.1: 𒁹𒀭𒀀𒋩𒊑𒅎𒉌𒊺𒋗

transl.: md a-šur3-re-em-ni-še-šu

HMM: {m}-{d}-a-šir ri-im-ni še-šu-

MEMM: {m}-{d}-a-nun-ri-im-ni še-šu

BiLSTM: {m}-{d}-a-ib-ri-im-ni še šu

l.2: 𒉺𒋼𒋛𒀭𒀀𒋩

transl.: ENSI2 d a-šur3

HMM: pa-te-si-{d}-a-šir

MEMM: pa-te si-{d}-a-nun-

BiLSTM: pa-{d}-a-li

l.3: 𒌉𒀭𒀀𒋩𒉌𒊏𒊑

transl.: DUMU d a-šur3-ne2-ra-ri

HMM: DUMU.{d}-a-šir ni ra-ri-

MEMM: DUMU {d}-a-nun-ni-ra-ri

BiLSTM: DUMU {d}-a-šir ni-ra-ri

l.4: 𒉺𒋼𒋛𒀭𒀀𒋩𒌉𒀭𒀀𒋩𒃲

transl.: ENSI2 d a-šur3 DUMU d a-šur3-GAL

HMM: pa-te-si-{d}-a-šir DUMU.{d}-a-šir GAL

MEMM: muš-te-si-{d}-a-nun-DUMU {d}-a-nun-GAL

BiLSTM: ENSI2 {d}-a-li DUMU {d}-a-LIL2 GAL

l.5: 𒉺𒋼𒋛𒂦𒊭𒁹𒆠𒆠𒀀𒁹𒄿𒆪𒉡𒌝

transl.: ENSI2 BAD3 ša mki-ki-a mi-ku-nu-um

HMM: pa-te-si-BAD3-ša {m}-{KI} {KI} a-{m}-i-ku-nu um-

MEMM: pa-te si-BAD3-ša {m}-ki-ki-a {m}-i-ku-nu ENSI2

BiLSTM: pa-BAD3 ša {m}-ki-KI.a {m}-i-ku-nu-um

l.6: 𒁹𒈗𒆤𒂗𒊭𒀭𒀀𒋩 (𒅤 [pu3] removed when given to program)

transl.: mLUGAL-ke-en pu3-zur8-d a-šur3

HMM: {m}-LUGAL {KI} EN.ša {d}-a-šir

MEMM: ana LUGAL ki-EN ša {d}-a-nun-

BiLSTM: diš LUGAL-ki-EN ša {d}-a-li

l.7: 𒁹𒀭𒀀𒋩𒉌𒊏𒊑𒌉𒅖𒈨𒀭𒁕𒃶

transl.: md a-šur3-ne2-ra-ri DUMU iš-me-d da-gan

HMM: {m}-{d}-a-šir ni ra-ri-DUMU.iš-MEŠ {d}-da-kan

MEMM: {m}-{d}-a-nun-ni-ra-ri-DUMU.iš-me-an-da-ar

BiLSTM: {m}-{d}-a-šir ni-ra-ri DUMU iš-me-an-da-kan

l.8: 𒀊𒁀𒅀𒌑𒌒𒁉𒋗𒉌𒂊𒈾𒄴𒈠

transl.: ab-ba-ia u2-up-pi2-šu-ni e-na-aḫ-ma

HMM: ap-ba-ia u2-ub bi-šu-ni e-na ih-ma

MEMM: ab-ba-ia u2-šek2-bi-šu ne2-e na-ah-ma

BiLSTM: ab-ba-ia u2-še-bi-šu-ni e-na-ah-ma

l.9: [illegible]

transl.: a-na ba-la$_2$-ṭi$_2$-ia u$_3$ ša-la$_2$-am URU.KI-ia

HMM: a-na ba-lal-hi-ia u$_3$ ša lal-am-{URU}-{KI} ia

MEMM: a-na ba-ṣu hi-ia u$_3$ ša {GU$_4$}-am-{URU}-ki-ia

BiLSTM: a-na ba-uṣ-hi-ia u$_3$ ša-an-am URU ki-ia

l.10: [illegible]

transl.: iš-tu uš-še-šu a-di ša-ap-ti-šu

HMM: iš-tu uš-še-šu a-di ša ap-ti šu-

MEMM: iš-tu-uš-še-šu a-di ša ap-ti-šu

BiLSTM: iš-tu uš-še-šu a-di ša ap-ti-šu

l.11: [illegible]

transl.: e-pu-uš u$_3$ si$_2$-ka$_3$-ti-šu a-na aš-ri-ši$_2$-na

HMM: e-bu-uš-u$_3$ zi-ga-ti šu a-na ina ri-si-na

MEMM: e-pu-uš u$_3$ zi-ga-ti-šu a-na ina re-si-na

BiLSTM: e-pu-uš u$_3$ zi-ga-ti-šu a-na ina ri-si-na

l.12: [illegible]

transl.: u$_2$-te-er ru-ba-u$_2$ ur-ki-u$_2$

HMM: u$_2$-te-ir-ru-ba-u$_2$-ur-{KI} u$_2$-

MEMM: u$_2$-te ir ru-ba-u$_2$-ur-qi$_2$-u$_2$-

BiLSTM: u$_2$-te-ir-ru ba u$_2$-liq-ke-u$_2$

l.13: [illegible]

transl.: e-nu-ma BAD$_3$ šu-ut e-nu-ḫu-ma

HMM: e-nu ma BAD$_3$-šu-tu$_2$ e-nu-hu-ma

MEMM: e-nu-ma sat-šu-ut SAG-nu-hu-ma

BiLSTM: e-nu-ma BAD$_3$ šu-tu$_2$ e-nu-hu-ma

l.14: [illegible]

transl.: e-ep-pu-šu ^{d}a-šur$_3$ u$_3$ ^{d}IŠKUR

HMM: e-ip-bu-šu-{d}-a-šir u$_3$ {d}-im-

MEMM: e-ep-pu-šu-{d}-a-nun-u$_3$ {d}-IŠKUR

BiLSTM: e-ip-pu-šu {d}-a-šir u$_3$ {d}-IŠKUR

l.15: [illegible]

transl.: ik-ri-bi-šu i-ša-me$_2$-u$_2$-šu

HMM: ik-ri-bi-šu-i-ša mi-u$_2$-šu-

MEMM: ik-ri-bi-šu i-ša mi-u$_2$-šu-

BiLSTM: ik-ri-bi-šu i-ša mi-u$_2$-šu-

l.16: [illegible]

transl.: si$_2$-ka$_3$-ti-šu a-na aš-ri-ši$_2$-na

HMM: zi-ga-ti šu a-na ina ri-si-na

MEMM: zi-ga-ti-šu a-na ina re-si-na

BiLSTM: zi-ga-ti-šu a-na ina ri-si-na

l.17: [illegible]

transl.: lu-te-er

HMM: lu-te-ir-

MEMM: lu-te ir

BiLSTM: lu-te-ir

A Middle-Babylonian *kudurru* (boundary stone) Royal Inscription of Nebuchadnezzar I, ca. 1125-1104, found in Sippar (Q006251)

l.1: 𒂊𒉡𒈠𒀭𒀝𒆪𒆪𒊑 𒉣𒈾𒀀𒁺 ([URU$_3$] removed since unknown)

transl.: e-nu-ma ^{d}AG-ku-dur$_2$-ri-URU$_3$ NUN na-a-du

HMM: e-nu ma {d}-ak-ku-DUR$_2$.ri-NUN na a-du-

MEMM: e-nu-ma {d}-AG ku-tal ri-NUN na-a du-

BiLSTM: e-nu-ma {d}-AG ku-dur$_2$-ri NUN na-a-du

l.2: 𒈾𒀾𒄣𒍢𒀉𒁷𒌁𒆠𒂊𒋼 𒈗𒈨𒌍

transl.: na-as-qu ṣi-it TIN.TIR.KI e-tel LUGAL.MEŠ

HMM: na-as-qu-ṣe-it-TIN.TIR {KI} e-til LUGAL.MEŠ

MEMM: na-as-qu ṣi-it-NIBRU {KI} e tel LUGAL.MEŠ

BiLSTM: na-aṣ-qu ṣi-it TIN.TIR {KI} e-tel-LUGAL.MEŠ

l.3: 𒉺𒋼𒋛𒃼𒁺𒄊𒀴𒌷𒄭

transl.: ENSI$_2$ qar-du GIR$_3$.NITA$_2$ URU.DUG$_3$

HMM: pa-te-si-qar-du GIR$_3$.NITA$_2$ {URU}-hi-

MEMM: pa-te si-qar-du GIR$_3$.NITA$_2$.{URU}-hi-

BiLSTM: pa-te-qar-du GIR$_3$.NITA$_2$ {URU}-hi

l.4: 𒀭𒌓𒆳𒋗𒈬𒊮𒈪𒄷𒉌𒅆𒋗

transl.: ^{d}UTU KUR-šu mu-šam-mi-ḫu ni-ši-šu

HMM: {d}-tu$_2$ {KUR}-šu-mu-u$_2$-mi-hu-ni ši-šu-

MEMM: {d}-UTU KUR šu-mu-šam-mi-hu-ni-ši-šu-

BiLSTM: NA$_4$ UTU KUR-šu-mu u$_2$ mi-hu-ne$_2$-ši šu-

l.5: 𒈾𒍣𒆪𒆪𒊑𒋾𒈬𒆸𒉡𒀊𒇷𒂊

transl.: na-ṣir ku-dur$_2$-re-ti mu-kin-nu AB-le-e

HMM: na ṣir ku-DUR$_2$.ri-ti mu-kin nu ap-li-e-

MEMM: na-muš-ku-tal ri-ti mu-kin-nu ap-le-e

BiLSTM: na-muš-ku-dur$_2$-ri-ti mu-kin-nu ap-le-e

l.6: 𒈗𒆠𒈾𒀀𒋾𒊭𒁲𒅔𒈪𒊺𒊑𒄿𒁷𒉡

transl.: LUGAL ki-na-a-ti ša di-in mi-ša$_2$-ri i-din-nu

HMM: LUGAL ki-na-a-ti ša di in-mi-ša$_2$ ri-i-TIN.nu

MEMM: LUGAL ki-na-a-ti ša di-in-mi-ša$_2$ ri-i-tu$_2$-nu

BiLSTM: LUGAL qi$_2$-na-a-ti ša-di-in-mi-ša$_2$-ri i din-nu-

l.7: 𒍣𒅅𒊒𒃼𒁺𒊺𒀀𒈾𒂊𒉺𒀀𒋫𒆠𒌅𒁕𒂊𒈬𒋡𒋙

transl.: zi-ik-ru qar-du ša$_2$ a-na e-peš ME$_3$ kit-pu-da e-mu-qa-šu$_2$

HMM: zi-ik-ru-qar-du-ša$_2$ a-na e-ger-ME$_3$ kit-bu-da-e-mu-qa-šu$_2$

MEMM: zi-ik-ru-qar-du ša$_2$ a-na e-muq NIBRU bu-da-e-mu-qa-šu$_2$

BiLSTM: zi ik-ru qar-du ša$_2$ a-na e-peš ME$_3$ sah-GID$_2$.DA e-mu qa-šu$_2$

l.8: [illegible]

transl.: na-aš$_2$ GIŠ.BAN ez-ze-ti la a-di-ru ME$_3$

HMM: na aš$_2$-{GIŠ}-PAN {GIŠ}-zi-ti la a-di ru-ME$_3$

MEMM: na-aš$_2$-{GIŠ}-ESI ez-zi-ti la-a-di ru-uh

BiLSTM: na-aš$_2$ {GIŠ}-PAN ez-ze-ti la a-di-ru ME$_3$

l.9: [illegible]

transl.: ša dan-na KUR.lul-lu-bi-i u$_2$-šam-qi$_2$-tu i-na GIŠ.TUKUL

HMM: ša reb na {KUR}-lip-lu-bi-i-u$_2$-šam-qi$_2$-tu i-na {GIŠ}-ku-

MEMM: ša dan-na {KUR}-lul-lu-bi i-u$_2$-šam-ki-tu i-na {GIŠ}-TUKUL

BiLSTM: ša dan-na {KUR}-lul-lu-bi-i-u$_2$-šam-qi$_2$-tu i-na {GIŠ}-TUKUL

l.10: [illegible]

transl.: ka-šid KUR a-mur-ri-i ša-li-lu kaš-ši-i

HMM: ka-šit-{KUR}-a-har-ri-i-ša li-lu-bi-ši i-

MEMM: ka-šid {KUR}-a-hur-ri-i-ša li-lu-bi-ši-i-

BiLSTM: ka-šid {KUR}-a-mur-ri-i ša li-lu-bi-ši i-

l.11: [illegible]

transl.: na-siq LUGAL.MEŠ NUN na-ra-am ^{d}AMAR.UTU

HMM: na siq-LUGAL.MEŠ NUN na ra-am-{d}-AMAR.tu$_2$

MEMM: na sik-LUGAL.MEŠ NUN na-ra-am-{d}-AMAR.UTU

BiLSTM: na SIG LUGAL.MEŠ NUN.na-ra-am {d}-AMAR.UTU

l.12: [illegible]

transl.: u$_2$-ta-ʾe-er-šu-ma LUGAL DINGIR.MEŠ ^{d}AMAR.UTU

HMM: u$_2$-ta-aʾ-ir-šu-ma LUGAL DINGIR.MEŠ {d}-AMAR.UTU

MEMM: u$_2$-ta-ʾu-ir-šu-ma UKKIN DINGIR.MEŠ {d}-AMAR.UTU

BiLSTM: u$_2$-ta-ʾi-ir-šu-ma LUGAL DINGIR.MEŠ {d}-AMAR.UTU

l.13: [illegible]

transl.: a-na tur-ri gi-mil-li KUR-URI.KI u$_2$-šat-ba-a GIŠ.TUKUL.MEŠ-šu

HMM: a-na DUMU.ri-gi-mil-li KUR URI {KI} u$_2$-šat-ba-a {GIŠ}-TUKUL.MEŠ šu

MEMM: a-na DUMU ri-gi-iš-li-{KUR}-URI {KI} u$_2$-KUR-ba-a-{GIŠ}-TUKUL.MEŠ-šu-

BiLSTM: a-na tur-ri-gi-mil-li KUR URI {KI} u$_2$-šat-ba-a {GIŠ}-TUKUL.MEŠ šu-

l.14: [illegible]

transl.: iš-tu URU.de-e-er ma-ḫa-az ^{d}a-nim

HMM: iš-tu {URU}-di e-ir-ma ha-aṣ {d}-a-ELAM.

MEMM: iš-tu {URU}-di-e ir ma-ha-as-{d}-a-nun-

BiLSTM: iš-tu {URU}-de-e-ir-ma-ha-aṣ {d}-a-num

l.15: [illegible]

transl.: ši-iḫ-ṭa iš-ta-ka-an a-na 30 KASKAL.GID$_2$

HMM: ši-ih-da-iš-ta-ka-an a-na KASKAL.bu

MEMM: ši-ih-da-iš-ta-ka-{d}-a-na eš-KASKAL.GID$_2$

BiLSTM: ši-ih-da-iš-ta-ka-{d}-a-na 30 KASKAL.GID$_2$

l.16: [illegible]

transl.: i-na ITI.<<ITI>>.ŠU.NUMUN.NA iṣ-ṣa-bat ḫar-ra-a-na

HMM: i-na {ITI}-{ITI}-šu-kul-na {GIŠ}-ṣa-be-mur-ra-a-na

MEMM: i-na uz-ITI šu kul-na-{GIŠ}-za-be-hur-ra-a-na

BiLSTM: i-na {ITI}-ITI šu NUMUN.na iṣ-ṣa-mit-har-ra a-na

l.17: [illegible]

transl.: TA kal ⸢RI⸣ aq-qu-ul-lu i-kab-ba-bu ki-i i-ša$_2$-ti

HMM: ta-reb ri-ak-qu-ul-lu-i-kab-ba-bu-{KI} i-i-ša$_2$ ti

MEMM: ta-kal-ri aq-qu-ul-lu-i-kap-ba-bu-ki-i i-ša$_2$ ti

BiLSTM: ta-dan-ri aq-qu ul-lu i-kab-ba-bu ki-i i-ša$_2$-ti

l.18: [illegible]

transl.: u$_3$ ṭu$_2$-⸢du?⸣ ša$_2$ ger-re-e-ti i-ḫa-am-ma-ṭu ki nab-li

HMM: u$_3$ tu du-ša$_2$ ger-re-e-ti i-ha-am-ma ṭu-{KI} nab-li-

MEMM: u$_3$ tu-du ša$_2$ ger-re-e-ti i-ha-am-ma-ṭu-ki-lal-li-

BiLSTM: u$_3$ tu-du-ša$_2$ ger-re-e-ti i-ha-am-ma-ṭu qi$_2$ nap-li-

l.19: [illegible]

transl.: ia-aʾ-nu A.MEŠ saḫ-ḫi u$_3$ bu-ut-tu-qu maš-qu-u$_2$

HMM: ia aʾ-nu MEŠ kit-hi-u$_3$ bu-tu$_2$ tu qu-maš-qu-u$_2$-

MEMM: ia ʾa-nu a-šib BARA$_2$.kit-hi u$_3$ pu-ut tu qu-maš-qu-u$_2$-

BiLSTM: ia-ʾa-nu A.MEŠ kit-hi u$_3$ pu-ut-tu-qu maš-qu u$_2$-

l.20: [illegible]

transl.: ni-is-qu ša$_2$ GAL.MEŠ ANŠE.KUR.RA.MEŠ it*-ta-ši-iz-zu

HMM: ni {GIŠ}-qu-ša$_2$ GAL.MEŠ ANŠE.KUR.RA.MEŠ it-ta-ši-{GIŠ}-zu-

MEMM: ni {GIŠ}-qu ša$_2$ GAL.MEŠ ANŠE.KUR.RA.MEŠ it-ta-ši-{GIŠ}-zu-

BiLSTM: ni-is-qu ša$_2$ GAL.MEŠ ANŠE.KUR.RA.MEŠ it-ta-ši-{GIŠ}-zu

l.21: [illegible]

transl.: u$_3$ ša$_2$ eṭ-li qar-di pu-ri-da-šu it-tu-ra

HMM: u$_3$ ša$_2$ it-li-qar-di bu-ri-da-šu-it-tu ra-

MEMM: u$_3$ ša$_2$ it-li-qar-di pu-ri-da-šu it-tu-ra-

BiLSTM: u$_3$ ša$_2$ it-li qar-di-bu re-da-šu it-tu-ra

l.22: [illegible]

transl.: il-lak LUGAL na-as-qu DINGIR.MEŠ na-šu-šu

HMM: il-šit-LUGAL na-as-qu-MEŠ na šu-šu-

MEMM: il-šiṭ-LUGAL na-as-qu DINGIR.MEŠ na-šu-šu

BiLSTM: il-lak LUGAL na-as-qu DINGIR.MEŠ na-šu-šu

l.23: [illegible] ([URU$_3$] removed since unknown)

transl.: i-red-di ^{d}AG-ku-dur$_2$-ri-URU$_3$ GABA.RI NU TUK

HMM: i-ret di {d}-ak-ku-DUR$_2$.ri-gap-ri-nu tuk-

MEMM: i-šem-di {d}-AG ku-tal ri gap-ri-nu tuk-

BiLSTM: i-rit-di {d}-AG ku-dur$_2$-ri GABA.ri nu-TUK

l.24: [illegible]

transl.: ul id-dar dan-na-at A.ŠA$_3$ iš-ka-ra-a-ti ul-lap

HMM: ul-it-DAR.reb na at-a-lib$_3$-iš-ka-ra-a-ti ul-reb

MEMM: ul-it-tak-lab-na-at a-ŠA$_3$.iš-ka-ra-a-ti ul-ziz

BiLSTM: ul id-mid dan-na-ad-a ŠA$_3$ iš ka-ra-a-ti ul-tan

l.25: [illegible]

transl.: ^{m}šit-ti-^{d}AMAR.UTU EN E$_2$ ša E$_2$-^{m}kar-zi-ia-ab-ku

HMM: {m}-šit-ti {d}-AMAR.tu$_2$ EN.E$_2$.ša E$_2$.{m}-te-a-zi-ia ap-ku-

MEMM: {m}-SANGA-ti {d}-AMAR.UTU EN-E$_2$ ša E$_2$-{m}-kar-zi-ia ap-ku-

BiLSTM: ana SANGA-ti {d}-AMAR.UTU EN E$_2$-ša E$_2$ {m}-kar-zi-ia-ap-ku

l.26: [illegible]

transl.: ša GIŠ.ma-gar-ra-šu E$_2$ i-mit-ti

HMM: ša {GIŠ}-ma ša$_2$ ra-šu-E$_2$.i-be-ti

MEMM: ša {GIŠ}-ma-ša$_2$-ra-šu-E$_2$ i-bat-ti

BiLSTM: ša {GIŠ}-ma ša$_2$-ra-šu-E$_2$ i-bat-ti

l.27: [illegible]

transl.: LUGAL EN-šu$_2$ la im-mer-ku-ma ma-gar-ra-šu$_2$ uk-til-la

HMM: LUGAL en-šu$_2$ la im-mir ku-ma ma ša$_2$ ra-šu$_2$ uk-til la

MEMM: LUGAL EN-šu$_2$ la-im-qut-ku-ma ma-ša$_2$-ra-šu$_2$ uk-TI.la-

BiLSTM: LUGAL EN-šu$_2$ la im-mir-ku-ma-ma gar-ra-šu$_2$-ug-til-la

l.28: [illegible]

transl.: ur-ri-iḫ-ma LUGAL dan-nu ik-ta-šad a-na GU$_2$ ID$_2$.u$_2$-la-a

HMM: ur-ri-ih-ma LUGAL dan-nu ik-ta-KUR a-na GUN a-u$_2$ la a-

MEMM: ur-ri ih-ma LUGAL dan-nu ik-ta-{KUR}-a-na-GU$_2$ {ID$_2$}-u$_2$-la-a-

BiLSTM: taš-ri-ih-ma LUGAL dan-nu iq-ta-{KUR}-a-na GU$_2$ {ID$_2$}-u$_2$-la-a

l.29: [illegible]

transl.: in-nen-du-ma LUGAL.MEŠ ki-lal-la-an ip-pu-šu ME$_3$

HMM: in-nen-du-ma LUGAL.MEŠ {KI} lal-la {d}-ip-bu-šu-ME$_3$

MEMM: in-nin du-ma LUGAL.MEŠ ki-lal-la-an-ep-pu-šu ṣib

BiLSTM: in-nen-du-ma LUGAL.MEŠ ki-lal-la-an ip-pu-šu ME$_3$

l.30: [illegible]

transl.: i-na bi-ri-šu-nu in-na-pi-iḫ i-ša$_2$-tu

HMM: i-na bi-ri-šu-nu in-na-pi-ih-i-ša$_2$-tu

MEMM: i-na be$_2$-ri-šu-nu in-na pi-ah-i-ša$_2$ tu

BiLSTM: i-na bi-ri-šu-nu in-na-pi-ih i-ša$_2$-tu

l.31: [illegible]

transl.: i-na tur-bu-ʾ-ti-šu-nu na-ʾ-du-ru IGI ^{d}UTU-ši

HMM: i-na DUMU.bu-aʾ-ti šu-nu na aʾ-du-ru-ši-{d}-tu$_2$ ši-

MEMM: i-na DUMU bu-ʾu ti-šu-nu na-ʾi-du-ru-ši-{d}-UTU-ši

BiLSTM: i-na DUMU bu-ʾu-ti šu-nu na-aʾ-du šub-ši-{d}-UTU-ši

l.32: [illegible]

transl.: a-šam-ša$_2$-tu iṣ-ṣa-nun-da i-sa-ar me-ḫu-u$_2$

HMM: a-u$_2$-ša$_2$ tu {GIŠ}-ṣa-NUN da-i-sa-ar-me-hu-u$_2$

MEMM: a-u$_2$ ša$_2$ tu {GIŠ}-za-NUN da-i-sa-ar-me hu-u$_2$-

BiLSTM: a u$_2$-ša$_2$-tu {GIŠ}-za-nun-da-i-sa-ar-me-hu-u$_2$

l.33: [illegible]

transl.: i-na me$_2$-ḫe-e ta-ḫa-zi-šu-nu

HMM: i-na mi-hi-e-ta-ha-zi-šu-nu

MEMM: i-na mi-ṣir e-ta-ha-zi-šu nu

BiLSTM: i-na mi-he-e ta-ha-zi-šu-nu

l.34: [illegible]

transl.: eṭ-lu EN GIŠ.GIGIR ul ip-pal-la-sa ša$_2$-na-a ša$_2$ it-ti-šu$_2$

HMM: it-lu-EN {GIŠ}-GIGIR.ul-ip-bal-la sa-ša$_2$ na-a ša$_2$ it-ti-šu$_2$

MEMM: it-lu-EN {GIŠ}-GIGIR.ul-ib-bal-la-sa-ša$_2$-na-a-ša$_2$-it-ti-šu$_2$

BiLSTM: eṭ-lu EN {GIŠ}-GIGIR ul ip-pal-la-sa ša$_2$-na-a ša$_2$ it-ti-šu$_2$

l.35: [illegible]

transl.: ^{m}šit-ti-^{d}AMAR.UTU EN E$_2$ ša$_2$ E$_2$-^{m}kar-zi-ia-ab-ku

HMM: {m}-šit-ti {d}-AMAR.tu$_2$ EN.E$_2$.ša$_2$ E$_2$.{m}-te-a-zi-ia ap-ku-

MEMM: {m}-SANGA-ti {d}-AMAR.UTU EN-E$_2$ ša$_2$ e$_2$-{m}-kar-zi-ia ap-ku-

BiLSTM: ana SANGA-ti {d}-AMAR.UTU EN E$_2$ ša$_2$-E$_2$ {m}-KAR-zi-ia ap-ku

l.36: [illegible]

transl.: ša GIŠ.ma-gar-<ma>-ra-šu E$_2$ i-mit-ti

HMM: ša {GIŠ}-ma ša$_2$ ma ra-šu-E$_2$.i-be-ti

MEMM: ša {GIŠ}-ma ša$_2$-ma ra-šu-E$_2$ i-bat-ti

BiLSTM: ša iz-ma ša$_2$ ma-ra-šu-E$_2$ i-bat-ti

l.37: [illegible]

transl.: LUGAL EN-šu$_2$ la im-mer-ku-ma GIŠ.ma-gar-ra-šu$_2$ uk-til-la

HMM: LUGAL en-šu$_2$ la im-mir ku-ma {GIŠ}-ma ša$_2$ ra-šu$_2$ uk-til la

MEMM: LUGAL EN-šu$_2$ la-im-qut-ku-ma {GIŠ}-ma-ša$_2$-ra-šu$_2$ uk-TI.la-

BiLSTM: LUGAL EN-šu$_2$ la im-mir-ku-ma {GIŠ}-ma ša$_2$-ra-šu$_2$-uk-til-la

l.38: [illegible]

transl.: la i-dur-ma ME$_3$ it-ta-rad a-na LU$_2$.KUR$_2$

HMM: la i-ṭur ma ME$_3$ it-ta-rat a-na {LU$_2$}-PAP.

MEMM: la i-du-ma ME$_3$-it-ta-GA a-na {LU$_2$}-x-

BiLSTM: la i-qam-ma ME$_3$ it-ta-rat a-na {LU$_2$}-KUR$_2$

l.39: [illegible]

transl.: u$_3$ a-na <LU$_2$>.KUR$_2$ EN-šu i-te-ru-ub a-tar-ta

HMM: u$_3$ a-na {LU$_2$}-PAP.EN.šu-i-te-ru-ub a-tar-ta

MEMM: u$_3$ a-na {LU$_2$}-x-EN šu i-te-ru-up a šil-ta-

BiLSTM: u$_3$ a-na {LU$_2$}-KUR$_2$ EN-šu i-te-ru-ub a-tar-ta-

l.40: [illegible]

transl.: i-na INIM ^{d}iš-tar u$_3$ ^{d}IŠKUR DINGIR.MEŠ EN.MEŠ ME$_3$

HMM: i-na ka-{d}-iš-tar-u$_3$ {d}-IŠKUR DINGIR.MEŠ EN.MEŠ ME$_3$

MEMM: i-na ka-{d}-iš-tar u$_3$ {d}-IŠKUR DINGIR.MEŠ EN.MEŠ-ni

BiLSTM: i-na KA {d}-iš-tar u$_3$ {d}-IŠKUR DINGIR.MEŠ EN.MEŠ ME$_3$

l.41: [illegible]

transl.: ul-te-es-ḫi-ir ḫul-te-lu-diš LUGAL KUR ELAM.MA.KI i-te-mid KUR-šu$_2$

HMM: ul-te-{GIŠ}-hi-ir-ši-ur-te-lu-{m}-LUGAL KUR ELAM.MA {KI} i-te-mid-{KUR}-šu$_2$

MEMM: ul-te {GIŠ}-hi-ir-ši-tiš$_2$ te lu-ana LUGAL KUR DAR.ma-ki-i te-ep-KUR-šu$_2$

BiLSTM: ul-te {GIŠ}-hi-ir-ši te-lu-ana LUGAL {KUR}-ELAM.MA {KI} i-te-ṣir KUR-šu$_2$

l.42: [illegible] ([URU$_3$] removed since unknown)

transl.: u$_3$ LUGAL ^{d}AG-ku-dur$_2$-ri-URU$_3$ it-ta-ši-iz i-na li-ti

HMM: u$_3$ LUGAL {d}-ak-ku-DUR$_2$.ri-it-ta-ši-iz i-na li-ti

MEMM: u$_3$ LUGAL {d}-AG ku-tal ri it-ta-ši {GIŠ}-i-na li-ti

BiLSTM: u$_3$ LUGAL DINGIR AG ku-dur$_2$-ri it-ta-ši-is i-na-li-ti

l.43: 𒅖𒍝𒁁𒆳𒉏𒈠𒆠𒄿𒅖𒋫𒇲𒃻𒂵𒊭

transl.: iṣ-ṣa-bat KUR ELAM.MA.KI i-iš-ta-lal NIG$_2$.GA-ša

HMM: {GIŠ}-ṣa-be-{KUR}-ELAM.ma {KI} i iš-ta-lal-ša$_2$ ga-ša

MEMM: {GIŠ}-za-be-{KUR}-kiš-ma-ki-i iš-ta-LAL-NIG$_2$.GA-ša

BiLSTM: iṣ-ṣa-mit {KUR}-ELAM.MA {KI} i iš-ta-lal NIG$_2$.GA ša

A Neo-Babylonian cylinder Royal Inscription of Nabonidus, ca. 551-542, found in Sippar (Q005422)

column i:

l.1: [illegible]

transl.: ^{d}na-bi-um-na-ʾi-⸢id⸣ LUGAL KA$_2$.DINGIR.RA.<KI>

HMM: {d}-na bi-um-na-aʾ-it-LUGAL KA$_2$.{d}-ra-{KI}

MEMM: {d}-na-bi-it na-ʾi-it-LUGAL KA$_2$.DINGIR.RA {KI}

BiLSTM: {d}-na-bi-um na-ʾi-id LUGAL KA$_2$.DINGIR.RA {KI}

l.2: [illegible]

transl.: SIPA ki-num li-pit ŠU.II ^{d}e$_2$-a

HMM: pa-lu-{KI} ELAM.li-E$_2$.šu-ia {d}-E$_2$.a-

MEMM: bar$_2$-sipa ki-lal-li-E$_2$-ŠU.II {d}-e$_2$-a

BiLSTM: SIPA ki-nim li-pit ŠU.II {d}-e$_2$-a

l.3: [illegible]

transl.: GURUŠ šu-pu$_2$-u$_2$ bi-nu-tu ^{d}nin-men-na

HMM: GURUŠ šu-PU$_2$.u$_2$-bi-nu tu {d}-nin-men-na

MEMM: EN šu-lum u$_2$-bi-nu-tu {d}-nin-men-na

BiLSTM: raš-šu SIG$_4$ u$_2$-bi-nu-tu {d}-nin-men-na

l.4: [illegible]

transl.: LUGAL la ša$_2$-na*-an* mi-gir ^{d}EN u$_3$ ^{d}AMAR.UTU

HMM: LUGAL la ša$_2$ na {d}-mi-ger-{d}-EN u$_3$ {d}-AMAR.tu$_2$

MEMM: LUGAL-la ša$_2$-na-an mi-ger-{d}-EN u$_3$ {d}-AMAR.UTU

BiLSTM: LUGAL la ša$_2$-na-an mi-gir {d}-EN u$_3$ {d}-AMAR.UTU

l.5: [illegible]

transl.: ma-al-ka it-pe-šu ni-bit ^{d}30 u ^{d}UTU

HMM: ma al-ka-it-pi-šu-ni E$_2$ {d}-30 u {d}-tu$_2$

MEMM: ma-al-ka-it-pi-šu ni-tum {d}-30 u {d}-UTU

BiLSTM: ma-al-ka it-pe-šu ni E$_2$ {d}-30 u {d}-UTU

l.6: [illegible]

transl.: NUN mu-un-dal-ka pa-li-iḫ DINGIR u$_3$ ^{d}15

HMM: NUN mu-un ri-ka-pa-li-ih DINGIR u$_3$ {d}-u

MEMM: NUN mu-un ri ka-pa-li-tum {d}-u$_3$ {d}-15

BiLSTM: NUN mu-un-ri-ka pa-eh {d}-u$_3$ {d}-15

l.7: [illegible]

transl.: GIR$_3$*.NITA$_2$* la-ni-i-ḫu mu-ṭi-ib ŠA$_3$ DINGIR GAL.GAL

HMM: GIR$_3$.NITA$_2$ la ni i-hu-mu-di ip-lib$_3$-{d}-GAL GAL

MEMM: GIR$_3$.NITA$_2$.la-ni-i hu-mu-di ŠA$_3$.{d}-GAL GAL

BiLSTM: GIR$_3$.ARAD-la ni-i-hu-mu-ṭe-ib ŠA$_3$.diŋir GAL.gal-

1.8: [illegible]

transl.: za-ni-in e$_2$-sag-il$_2$ u$_3$ e$_2$-zi-da

HMM: ṣa-ni in-e$_2$-sag-il$_2$ u$_3$ E$_2$.zi-da-

MEMM: za-ni in-e$_2$-sag-il$_2$ u$_3$ E$_2$ zi-da

BiLSTM: za-ni-in e$_2$-sag-il$_2$ u$_3$ e$_2$-zi-da

1.9: [illegible]

transl.: mu-ud-diš ma-ḫa-zu mu-šak-lil eš-re-e-ti

HMM: mu-tu$_2$ {m}-ma ha-zu-mu-šak-lil eš-re-e-ti

MEMM: mu-tu$_2$ {m}-ma-ha-zu mu-šak-lil eš-re-e-ti

BiLSTM: mu-ud-diš ma-ha-zu mu-šak-lil eš-re-e-ti

1.10: [illegible]

transl.: ENSI$_2$ ṣi-i-ri mu-ṭaḫ-ḫi-id sat-tuk-ku

HMM: pa-te-si-ṣe-i-ri-mu-tah-hi-it-sat-tuk-ku

MEMM: pa-te si-ṣi-i-ri mu-ṭah-hi-it-{KUR}-tuk-ku-

BiLSTM: pa-te-ṣi-i-ri mu-tah-hi-et sat-tuk-ku

1.11: [illegible]

transl.: ša$_2$ a-na pa-la-aḫ DINGIR.MEŠ pi-it-qu-du

HMM: ša$_2$ a-na pa-la-ah DINGIR.MEŠ pi-it-qu-du-

MEMM: ša$_2$ a-na pa-la-aṣ DINGIR.MEŠ pi-it-qu-du

BiLSTM: ša$_2$ a-na pa-la-ah DINGIR.MEŠ pi iṭ-qu-du

1.12: [illegible]

transl.: la ig-gu-u$_2$ mu-ši u$_3$ ur-ra

HMM: la ik-gu-u$_2$-mu-ši-u$_3$ ur-ra-

MEMM: la-ik-gu-u$_2$-mu-ši-u$_3$ ur-ra

BiLSTM: la ig-gu-u$_2$ mu-ši u$_3$ taš-ra

1.13: [illegible]

transl.: DUMU ^{md}AG-TIN-su-iq-bi ⸢NUN⸣ em$_4$-qa$_2$ a-na-ku

HMM: DUMU {m}-{d}-ak-TIN.su-ik-bi-NUN EME.ga-a-na-ku

MEMM: DUMU {m}-{d}-AG-TU su-ik-bi NUN EGIR-GA a-na ku

BiLSTM: DUMU {m}-{d}-AG-TIN-su-iq-bi NUN MURUB$_4$-GA a-na-ku

1.14: [illegible]

transl.: a-na ^{d}UTU EN ⸢ra⸣-[bu]-u$_2$ be-li-ia

HMM: a-na {d}-tu$_2$ EN.ra-bu-u$_2$-be-li-ia

MEMM: a-na {d}-UTU EN-ra-bu-u$_2$-bat-li-ia

BiLSTM: a-na {d}-UTU EN ra-pu-u$_2$-be-li-ia

1.15: [illegible]

transl.: u$_3$ ^{d}a-a kal-⸢la⸣-[tum] be-el-tum GAL-tum

HMM: u$_3$ {d}-a-a-reb la tum be-el-tum GAL tum

MEMM: u$_3$ {d}-a-a KAL.la tum be-let tum GAL.BI.

BiLSTM: u$_3$ AN.a a dan-la-tum be-el-tum GAL-tum

l.16: [illegible]

transl.: be-el-<ti>-ia* uš-te-mi-iq-ma

HMM: be-el-ti-ia uš-te-mi-ik-ma

MEMM: LUL el-ti-ia uš-te-mi-ik-ma

BiLSTM: be-el-ti-ia uš-te-mi-iq-ma

l.17: [illegible]

transl.: e$_2$-babbar-ra E$_2$-su-un ina qe$_2$-reb ZIMBIR.KI

HMM: E$_2$.tu$_2$ ra-bit-su-un ina {KI} reb tu$_2$ kib-NUN {KI}

MEMM: e$_2$-šar$_2$-ra E$_2$ su-un ina qe$_2$-reb ZIMBIR {KI}

BiLSTM: e$_2$-par-ra bit-su-un ina qe$_2$-reb ZIMBIR {KI}

l.18: [illegible]

transl.: e-li te-me-en na-ram-^{d}30 LUGAL ul-la

HMM: e-li-te-MEŠ EN.na ram-{d}-LUGAL ul-la

MEMM: e li-te me-en-na-ram {d}-30 LUGAL ul-la-

BiLSTM: e-li te-me-en-na-ram {d}-30 LUGAL ul-la

l.19: [illegible]

transl.: e-eš-ši-iš u$_2$-še-pi-iš-ma

HMM: e-ši-iš u$_2$-še-pi-iš-ma

MEMM: e-30 LIM iš-u$_2$-še-pi-iš-ma

BiLSTM: e-eš-ši-iš u$_2$-še-pi-iš-ma

l.20: [illegible]

transl.: a-na ta-na-da-a-ti aš$_2$-tak-ka-an

HMM: a-na ta-na-da-a-ti aš$_2$-tak-ka-an

MEMM: a-na ta-na-da-a-ti aš$_2$-tak-ka-{d}-

BiLSTM: a-na ta-na-da-a-ti aš$_2$-tak-ka-an

l.21: [illegible]

transl.: a-na ⸢ši-pu-šu$_2$⸣ GIŠ.GIŠIMMAR dan-nu-tu u$_2$-šar-ši-id

HMM: a-na ši-bu-šu$_2$ {GIŠ}-SA$_6$ reb nu tu u$_2$-šar-ši-it-

MEMM: a-na ši-bu-šu$_2$ {GIŠ}-IG.dan-nu tu u$_2$-šar-ši it-

BiLSTM: a-na IGI-bu-šu$_2$ {GIŠ}-NA$_2$ dan-nu-tu u$_2$-šar-ši-it

l.22: [illegible]

transl.: GIŠ.EREN pa-ag-lu-tu tar-bit KUR.ḫa-ma-ni

HMM: {GIŠ}-EREN pa-ak-lu-tu tar-bit {KUR}-ha-ma ni

MEMM: {GIŠ}-EREN pa-aq-lu-tu tar-bit {KUR}-ha-ma-ni

BiLSTM: {GIŠ}-EREN pa aq-lu-tu tar-bit {KUR}-ha-ma-ni

l.23: [illegible]

transl.: u$_3$ KUR.lab-<na>-ni* a-na ṣu-lu-li-šu

HMM: u$_3$ {KUR}-reb na ni a-na ṣu-lu-li-šu-

MEMM: u$_3$ {KUR}-lab-na-ni a-na ṣu-lu-li-šu-

BiLSTM: u$_3$ {KUR}-lab-na-ni a-na ṣu-lu-li-šu-

l.24: [illegible]

transl.: u GIŠ.IG.MEŠ KA$_2$.MEŠ-šu u$_2$-šat-mi-iḫ

HMM: u {GIŠ}-IG.MEŠ KA$_2$.MEŠ eš-šu-u$_2$-{KUR}-mi-ih-

MEMM: u {GIŠ}-IG.MEŠ KA$_2$.MEŠ-šu u$_2$-šat-mi-eh-

BiLSTM: u {GIŠ}-IG.MEŠ KA$_2$.MEŠ-šu-u$_2$ šat-mi-eh

l.25: [illegible]

transl.: u$_2$-nu-tu-šu ina KU$_3$.BABBAR u KU$_3$.GI u$_2$-za-ʾi-in-ma

HMM: u$_2$-nu tu šu-ina KU$_3$.tu$_2$ u KU$_3$.gi-u$_2$-ṣa-aʾ-in-ma

MEMM: u$_2$-nu-tu šu-ina KU$_3$.BABBAR u KU$_3$.GI u$_2$-za-ʾi-in-ma

BiLSTM: u$_2$-nu-tu-šu ina KU$_3$.BABBAR u KU$_3$.GI u$_2$-za-ʾi-in-ma

l.26: [illegible]

transl.: a-na tab-ra-a-tu$_2$ la-la-a uš-mal$_2$-la

HMM: a-na tab-ra-a-tu$_2$ la la a-uš-si-a-la

MEMM: a-na tap-ra-a-tu$_2$ la-la-a-si-a la

BiLSTM: a-na tab-ra-a-tu$_2$ la-la-a uš-ṭir la

l.27: [illegible]

transl.: ši-ṭir MU-ia KI ši-ṭir šu-mu ša$_2$ na-ram-^{d}30

HMM: ši-si-a-mu-ia {KI} ši-si-a-šu-mu-ša$_2$ na ram-{d}-

MEMM: ši-si-a-mu-ia ki-ši-si-a-šu mu-ša$_2$-na-ram {d}-30

BiLSTM: ši-ṭir MU-ia qi$_2$ ši ṭir šu-mu ša$_2$ na-ram {d}-30

l.28: [illegible]

transl.: LUGAL maḫ-ru aš$_2$-tak-kan qe$_2$-er-bi-uš-šu

HMM: LUGAL mah-ru-aš$_2$-tak-kan-{KI} ir-bi-uš-šu-

MEMM: LUGAL mah-ru-aš$_2$-pur kan-ki-ir bi-uš-šu

BiLSTM: LUGAL mah-ru aš$_2$-tak-HE$_2$.ki-er-bi-uš-šu

l.29: [illegible]

transl.: E$_2$ ša$_2$ ^{d}UTU u ^{d}a-a EN.MEŠ-a ina im-na u ⸢šu$_2$-me-lu⸣ ki-ma u$_4$-me

HMM: E$_2$.ša$_2$ {d}-UTU u {d}-a-a-EN.MEŠ a-ina im-na u šu$_2$ MEŠ lu-{KI} ma tu$_2$ MEŠ

MEMM: E$_2$-ša$_2$ an-E$_3$ {d}-a-a EN.MEŠ a-ina {IM}-na-MAN.šu$_2$ me lu-ki-ma u$_4$-me

BiLSTM: E$_2$ ša$_2$ {d}-UTU u {d}-a-a EN.MEŠ-a ina im-na u šu$_2$ me-lu ki-ma u$_4$-me

l.30: [illegible]

transl.: uš-⸢nam-mir⸣-ma e$_2$-kur-ra E$_2$ ^{d}bu-ne-ne ša$_2$ ⸢qe$_2$-reb⸣ si-par

HMM: uš-nam-mir ma E$_2$.{KUR}-ra-E$_2$.{d}-bu-ṭe$_3$-ṭe$_3$-ša$_2$ {KI} reb si-ut

MEMM: uš-NAM TUKUL-ma e$_2$-šar$_2$-ra E$_2$ {d}-bu-biš nap-ša$_2$ qe$_2$-reb si par

BiLSTM: uš-nam-mir ma e$_2$-kur-ra E$_2$ {d}-bu-de$_3$ de$_3$ ša$_2$ qe$_2$-reb si-pir

l.31: [illegible]

transl.: ana ^{d}bu-ne-ne EN-ia$_2$ eš-šiš e-pu$_2$-uš*-ma* te-bi-ib-ti-šu$_2$

HMM: {m}-{d}-bu-ṭe$_3$-ṭe$_3$-EN.ŠEŠ.e-PU$_2$.uš-ma te-bi-ip-TI-šu$_2$

MEMM: {m}-{d}-bu-biš eh-EN.ia$_2$ eš-ŠEŠ-e mut-uš-ma-te be$_2$-ep-ti-šu$_2$

BiLSTM: diš {d}-{d}-NE ṭe$_3$-EN-ia$_2$ eš-šiš e-IBILA uš-ma te-bi ep-ti-šu$_2$

l.32: [illegible]

transl.: u$_2$-qa-ad-diš-ma us-si-ma ana E$_2$.KUR DINGIR-u$_2$-ti-šu$_2$ ki-ma u$_4$-me zi-mu-šu u$_2$-ša$_2$-an-na-bi-iṭ

HMM: u$_2$-qa-at-{m}-ma us-si-ma {m}-E$_2$.{KUR}-DINGIR-u$_2$-ti šu$_2$ {KI} ma tu$_2$ MEŠ zi-mu-šu-u$_2$-ša$_2$ {d}-na bi-it-

MEMM: u$_2$-qa-ad-{m}-ma-us-si-ma ana E$_2$-KUR {d}-šam-ti-šu$_2$ ki-ma u$_4$-me zi-mu-šu-u$_2$ ša$_2$ an-na-bi-it

BiLSTM: u$_2$-qa-at {m}-ma-us-si-ma ana E$_2$ KUR DINGIR-u$_2$-ti-šu$_2$ ki-ma u$_4$-me zi-mu-šu u$_2$-ša$_2$-an-na-bi-id

l.33: [illegible]

transl.: E$_2$.GAR$_8$.MEŠ ša$_2$ si-ḫir-tu$_2$ e$_2$-babbar-ra ša$_2$ KA$_2$ AŠ.AM$_3$

HMM: E$_2$.GAR$_8$.MEŠ ša$_2$ si-hir-tu$_2$ E$_2$.tu$_2$ ra-ša$_2$ KA$_2$.ina a-an

MEMM: E$_2$ AD-MEŠ ša$_2$ si-hir-tu$_2$ e$_2$-šar$_2$-ra ša$_2$ KA$_2$.ina a-{d}-

BiLSTM: E$_2$.GAR$_8$.MEŠ ša$_2$ si-hir-tu$_2$ e$_2$-par-ra ša$_2$ KA$_2$ AŠ.AM$_3$

l.34: [illegible]

transl.: u$_2$-še-piš-ma UGU ša$_2$ IGI u$_2$-dan-nin ši-ṭir šu-mi-ia$_2$

HMM: u$_2$-še-ger-ma u ka-ša$_2$ ši-u$_2$-dan-nin-ši-ṭir šu-mi-

MEMM: u$_2$-še-piš-ma UGU ša$_2$ ši-u$_2$-dan-nin-ši-si-a-šu-mi-ia$_2$

BiLSTM: u$_2$-še-piš-ma UGU ša$_2$-ši u$_2$-dan-nin ši-ṭir šu-mi-ia$_2$

l.35: [illegible]

transl.: u ṣa-lam LUGAL-u$_2$-ti-ia$_2$ ma-ḫar ^{d}UTU u$_3$ ^{d}a-a EN.MEŠ-u$_2$-a

HMM: u ṣa-lam LUGAL-u$_2$-ti ma har-{d}-UTU u$_3$ {d}-a-a-EN.MEŠ u$_2$-a-

MEMM: u ṣa-lam LUGAL-u$_2$-ti ia$_2$ ma har {d}-UTU u$_3$ {d}-a-a EN.MEŠ-u$_2$-a-

BiLSTM: u ṣa-lam LUGAL-u$_2$-ti-ia$_2$ ma-har {d}-UTU u$_3$ {d}-a-a EN.MEŠ u$_2$-a

column ii:

l.1: [illegible]

transl.: u$_2$-ki-in a-na du-ur u$_4$-mi

HMM: u$_2$-{KI} in a-na du-ur-tu$_2$ mi-

MEMM: u$_2$-ki-in-a-na du-ur-par mi-

BiLSTM: u$_2$-ki-in a-na du-ur u$_4$-mi

l.2: [illegible]

transl.: ^{d}UTU EN ṣi-i-ri ra-ʾi-im na-piš-tu$_2$

HMM: {d}-tu$_2$ EN.ṣe-i-ri-ra-aʾ-im-na ger-tu$_2$

MEMM: {d}-UTU EN-ṣi-i-ri ra-aʾ-im-na-qer-tu$_2$

BiLSTM: {d}-UTU EN ṣi-i-ri ra-aʾ-im na-piš-tu$_2$

l.3: [illegible]

transl.: a-na e$_2$-babbar-ra E$_2$-ka na-am-ra

HMM: a-na E$_2$.tu$_2$ ra-E$_2$.ka-na am-ra-

MEMM: a-na E$_2$ ud-ra E$_2$ ka-na-am-ra

BiLSTM: a-na e$_2$-par-ra e$_2$-ka-na-am-ra

l.4: [illegible]

transl.: ina a-ṣe-e-ka u$_3$ e-re-bi-ka

HMM: ina a ṣe-e-ka-u$_3$ e-ri-bi-ka-

MEMM: ina a-ṣe-e-ka-u$_3$ e-ri-bi ka-

BiLSTM: ina a-ṣe-e-ka u$_3$ e-ri-bi-ka-

l.5: [illegible]

transl.: ep$_2$-še-tu-u$_2$-a SIG$_5$-a-tu$_2$ ši-ṭir šu-mi-ia

HMM: tum še-tu u$_2$-a-ši-ERIM.a-tu$_2$ ši-ṭir šu-mi-ia

MEMM: tum še-tu-u$_2$-a-ši-pir-a-tu$_2$ ši-si-a-šu-mi-ia

BiLSTM: tum še-tu-u$_2$-a ši-a-tu$_2$ ši-ṭir šu-mi-ia

l.6: [illegible]

transl.: u$_3$ ṣa-lam LUGAL-u$_2$-ti-ia ḫa-di-iš

HMM: u$_3$ ṣa-lam LUGAL-u$_2$-ti-ia ha-di iš-

MEMM: u$_3$ ṣa-lam LUGAL-u$_2$-ti-ia ha-di iš-

BiLSTM: u$_3$ ṣa-lam LUGAL-u$_2$-ti-ia ha-di-iš

l.7: [illegible]

transl.: na-pa-lis-ma SIG$_5$-tu-u$_2$-a ana du-ur da-ri$_2$

HMM: na pa-liš ma ši-ERIM.tu u$_2$-a-{m}-du-ur-da-{URU}-

MEMM: na-pa-tiq ma ši ṣab-tu-u$_2$-a {m}-GIN-NUMUN da-{URU}-

BiLSTM: na-pa-lis-ma SIG$_5$-tu-u$_2$-a {m}-du-ur-da-{URU}-

l.8: [illegible]

transl.: lib-ša-aʾ-ma a-na maḫ-ri-ka

HMM: lip-ša aʾ-ma a-na mah-ri-ka-

MEMM: lul-ša ʾa-ma a-na mah-re-ka-

BiLSTM: nar-ša-iʾ-ma a-na mah-ri-ka

l.9: [illegible]

transl.: a-ra-ku UD.ME LUGAL-u$_2$-ti-ia liš-ša$_2$-kin ina pi-i-ka

HMM: a-ra-ku-tu$_2$ MEŠ LUGAL-u$_2$-ti ia liš-ša$_2$-kin ina pi-i-ka-

MEMM: a-ra-ku u$_4$-me LUGAL-u$_2$-ti-ia BAL-ša$_2$-qi-ina pi-i-ka-

BiLSTM: a-ra-ku u$_4$-me LUGAL-u$_2$-ti-ia liš-ša$_2$-kin ina pi-i-ka

l.10: [illegible]

transl.: ina nu-u$_2$-ri-ka na-am-⸢ri⸣ lu-la-ab-bi-ir

HMM: ina nu u$_2$-ri-ka-na am-ri-lu-la ap-bi-ir-

MEMM: ina nu-u$_2$-ri-ka-na-am-ri lu-la ab-bi ir-

BiLSTM: ina nu-u$_2$ ri-ka na-am-tal-lu-la-ab-bi ir-

l.11: [illegible]

transl.: tal*-lak-ka a-na ⸢da⸣-ra-⸢a⸣-ti*

HMM: ri-šit-ka a-na da-ra-a-ti

MEMM: ri šid-ka-a-na da-ra-a-ti

BiLSTM: ri lak-ka a-na da-ra-a-ti

l.12: [illegible]

transl.: li-ku-un BALA-lu-u$_2$-a ^{d}a-a

HMM: li-ku-un bal-lu-u$_2$-a {d}-a-a-

MEMM: li-ku-un bal-lu-u$_2$ a-{d}-a-a

BiLSTM: li-ku-un pal-lu-u$_2$-a a-a

l.13: [illegible]

transl.: kal-la-tum GAL-tum na-ram-mat ^{d}UTU-ši

HMM: reb la tum GAL tum na ram-{KUR}-{d}-tu$_2$ ši-

MEMM: KAL.la tum GAL.BI.na-ram KUR {d}-UTU-ši

BiLSTM: a-la-tum GAL-tum na-ram mat {d}-UTU-ši

l.14: [illegible]

transl.: ina e$_2$-ki-nu$_2$ E$_2$-ka nam-ri ḫa-diš ina a-ša$_2$-bi-ka

HMM: ina E$_2$.{KI} NA$_2$ E$_2$.ka-nam-ri-ha-{m}-ina a-ša$_2$ bi-ka-

MEMM: ina E$_2$ {KI} dil-E$_2$ ka-nam-ri ha-{m}-ina a-ša$_2$ bi ka-

BiLSTM: ina e$_2$-ki-AD bit ka nam-ri ha diš ina a-ša$_2$-bi-ka

l.15: [illegible]

transl.: ma-ḫar ^{d}UTU nu-ur$_2$ DINGIR su$_2$-pi-ia SIG$_5$.MEŠ

HMM: ma har-{d}-tu$_2$ nu ur$_2$-{d}-zu-pi-ia SIG$_5$.MEŠ

MEMM: ma-har {d}-UTU nu UR$_2$ {d}-zu-pi-ia ši ERIM.MEŠ

BiLSTM: ma-har an tu$_2$ nu-AM$_3$ {d}-zu-pi-ia SIG$_5$.MEŠ

l.16: [illegible]

transl.: šu-ri-ka u$_4$-mi ba-la-ṭi-ia

HMM: šu-ri-ka-tu$_2$ mi-ba-la di ia

MEMM: šu-ri ka-tu$_2$ mi-ba la di-ia

BiLSTM: šu-re-ka tu$_2$ mi ba-la-di-ia

l.17: [illegible]

transl.: suk-kal-lum mit-lu-ku dam-na ^{d}bu-ne-ne

HMM: AS$_4$.reb lum be-lu-ku-{d}-am-na {d}-bu-ṭe$_3$-ṭe$_3$-

MEMM: gul-KAL-lum be-lu-ku-{d}-am-na-an-pu-uk-1/2

BiLSTM: {KUR}-dan-lum be-lu-ku an-am-na-an-bu bil NE.

l.18: [illegible]

transl.: ša$_2$ mi-lik-šu dam-qa ma-ḫar ^{d}UTU u ^{d}a-a x x-⸢ka⸣

HMM: ša$_2$ mi-ur-šu-dam-qa-ma har-{d}-UTU u {d}-a-a x x ka-

MEMM: ša$_{2}$ mi-ur-šu-qaq-qa-ma-har {d}-UTU u {d}-a-a x x-ka-

BiLSTM: ša$_{2}$ mi-lik šu-dam-qa-ma-har {d}-UTU u {d}-a-a x x ka-

l.19: [illegible]xxx

transl.: a-ṣu$_{2}$-ka šu-lu-lu el-ṣi-iš ina x x x

HMM: a-zu-ka-šu-lu-lu-el-ṣe-iš-ina x x x

MEMM: a-zu-ka-šu-lu-lu-el-ṣi-iš-ina x x x

BiLSTM: a-zu-ka šu-lu-lu el-ṣi-iš ina x x x

l.20: [illegible]

transl.: ma-ḫar ^{d}UTU EN gim-ri šal-ṭiš ina ⸢u$_{2}$-zu-zi-ka⸣

HMM: ma har-{d}-tu$_{2}$ EN.GIM ri-šal-{m}-ina u$_{2}$-zu-zi-ka-

MEMM: ma-har {d}-UTU EN-GIM ri-is {m}-ina u$_{2}$-zu-zi-ka-

BiLSTM: ma-har {d}-UTU EN gim-ri {MUNUS}-ṭiš ina KUŠ$_{3}$ zu-zi-ka

l.21: [illegible]

transl.: e-ger$_{2}$-⸢re*⸣-e SIG$_{5}$-ti-⸢ia$_{2}$ lu⸣ sa-⸢dir ina KA⸣-ku

HMM: e-GIR$_{2}$ re-e-ši-ERIM.ti lu-sa-si-a-ina ka-ku-

MEMM: e-tel-re-e ši-pir-ti ia$_{2}$ lu-sa-si-a ina ka-ku-

BiLSTM: e šiṭ-re-e ši-ti-ia$_{2}$ lu-sa-dir a ina ka-ku

l.22: [illegible]

transl.: ina lib$_{3}$*-bi-šu MAḪ šal-mi-iš it-tal-lak

HMM: ina lib$_{3}$-bi-šu-mah-šal-mi-iš-it-ri-šit-

MEMM: ina lib$_{3}$-bi šu mah-MUNUS.mi-iš-it-ri-SANGA-

BiLSTM: ina lib$_{3}$-bi-šu-MAH šal-mi-iš ed-tal-lak

l.23: [illegible]

transl.: GIŠ.GU.ZA LUGAL-u$_{2}$-ti-ia$_{2}$ lu-lab-bir a-di še-be$_{2}$ lit-tu-tu

HMM: {GIŠ}-GU.ZA LUGAL-u$_{2}$-ti lu-reb bir-a-di še-bi-lit-tu tu

MEMM: {GIŠ}-GU.ZA LUGAL-u$_{2}$-ti ia$_{2}$ lu-KAL-bir-a-di še-bi-rit tu tu

BiLSTM: {GIŠ}-GU.ZA LUGAL-u$_{2}$-ti-ia$_{2}$ lu-kal-bir a-di še-bi lit-tu-tu

l.24: [illegible]

transl.: DINGIR.⸢MEŠ⸣ (over erasure) sip-par.KI u e$_{2}$-babbar-ra ma-ḫar ^{d}UTU

HMM: DINGIR.MEŠ MEŠ tu$_{2}$ {KI} u E$_{2}$.tu$_{2}$ ra-ma har-{d}-UTU

MEMM: DINGIR.MEŠ me-tum ki u e$_{2}$-šar$_{2}$-ra ma-har {d}-UTU

BiLSTM: DINGIR.MEŠ me-BABBAR-qi$_{2}$-u e$_{2}$-ut-ra ma-har {d}-UTU

l.25: [illegible]

transl.: u ^{d}a-a li-dam-qa ep$_{2}$-še-tu-u$_{2}$-a

HMM: u {d}-a-a-li-dam-qa-tum še-tu-u$_{2}$-a

MEMM: u {d}-a-a li DARA$_{3}$ qa-tum še-tu-u$_{2}$-a-

BiLSTM: u {d}-a-a li dam-qa-tum še-tu-u$_2$-a

l.26: [illegible]

transl.: a-na-ku lu-u$_2$ LUGAL da-ru-u$_2$ za-ni-in

HMM: a-na ku-lu-u$_2$-LUGAL da-ru-u$_2$-ṣa-ni in-

MEMM: a na-ku lu-u$_2$ LUGAL da-ru-u$_2$-ṣa-ni-in-

BiLSTM: a-na-ku lu-u$_2$ LUGAL da-ru u$_2$-za-ni-in

l.27: [illegible]

transl.: ⸢lu-pu⸣-uš bi-lat-⸢si⸣-nu ša$_2$ ka-liš kib-rat ma-ḫar ^{d}ŠU$_2$

HMM: lu-bu-uš-bi-{KUR}-si-nu ša$_2$ ka-liš kib-rat ma har-{d}-šu$_2$

MEMM: lu-pu-uš bi {KUR}-si-nu ša$_2$ ka-liš kib-rat LIMMU$_2$-hur-an-šu$_2$

BiLSTM: lu-pu-uš-bi {KUR}-si-nu ša$_2$ ka-liš kib-rat ma-har {d}-šu$_2$

l.28: [illegible]

transl.: u ^{d}zar-pa-ni-tum ^{d}AG u ^{d}U.GUR DINGIR.MEŠ-u$_2$-a u DINGIR gi-mir-šu$_2$-nu

HMM: u {d}-zar-pa-ni-tum {d}-AG u {d}-U.GUR DINGIR.MEŠ u$_2$-a-u {d}-gi-mir šu$_2$-nu

MEMM: u {d}-zar-pa-ni-tum {d}-AG u {d}-U.GUR DINGIR.MEŠ u$_2$-a-u {d}-gi-mir šu$_2$-nu

BiLSTM: u {d}-zar-pa-ni-tum {d}-AG u {d}-U.GUR DINGIR.MEŠ u$_2$-a u {d}-gi-mir-šu$_2$-nu

l.29: [illegible]

transl.: a-šib si-ḫir-tu$_2$ a$_2$-ki-it ša$_2$ LUGAL DINGIR ša$_2$-qu-u$_2$

HMM: a-MEŠ si-hir-tu$_2$ it-{KI} it-ša$_2$ LUGAL {d}-ša$_2$-qu-u$_2$-

MEMM: a-šib si-hir-tu$_2$ it-ki-it-ša$_2$ LUGAL {d}-ša$_2$-maš u$_2$-

BiLSTM: a-šib si-hir-tu$_2$ id-ki-it ša$_2$ LUGAL {d}-ša$_2$-qu-u$_2$

l.30: [illegible]

transl.: EN EN.EN ZAG.MUK re-eš šat-ti i-sin-nu a$_2$-ki-it

HMM: EN.EN.EN.zaq-muq re-eš šat-ti i-nu it-{KI} it-

MEMM: EN EN-EN-zak-gim-ri-eš-{KUR}-ti i-eš-nu ed-ki-it-

BiLSTM: EN EN.EN GURUŠ PAD.ri eš-šat-ti i-sin-nu id-qi$_2$ it

l.31: [illegible]

transl.: ana ni-qe$_2$-e ma-aṣ-ḫa-tu$_2$ u pa-qa-du e$_2$-⸢da-di⸣-ḫe$_2$-gal$_2$

HMM: {m}-ni ke-e-ma as-ha-tu$_2$ u pa-qa-du-E$_2$.da-di kan-ik-

MEMM: {m}-ni-ki-e-ma aṣ-ha-tu$_2$ u pa-qa-du-E$_2$ da-di HE$_2$.GAL$_2$

BiLSTM: {m}-ni-ke-e-ma as-ha-par-u pa-qa-du E$_2$ da-di HE$_2$.GAL$_2$

l.32: [illegible]

transl.: u$_3$ ut-ne$_2$-en-na EN EN.EN

HMM: u$_3$ tu$_2$ ni EN.na EN.EN.EN.

MEMM: u$_3$ ZABAR na EN.EN EN-

BiLSTM: u$_3$ tu$_2$-ni EN.NA EN EN.EN

l.33: 𒇻𒊓𒀜𒊏𒀝𒊑𒆷𒌈

transl.: lu sa-ad-ra-ak tal-lak-tum

HMM: lu-sa-at-ra-ak-ri-šit-tum

MEMM: lu-sa-ad-ra-ak-ri šid-tum

BiLSTM: lu-sa-aṭ-ra-ak-tal-lak-tum

l.34: 𒀀𒈾𒁕𒌷𒀀𒌅𒇷𒅕𒀀𒋗𒁄𒇻𒌑𒀀

transl.: a-na da-ri$_2$-a-tu$_2$ ⸢li-ir-a-šu⸣ BALA-lu-u$_2$-a

HMM: a-na da-{URU}-a-tu$_2$ li-ir-a-šu-bal-lu-u$_2$-a-

MEMM: a-na da-{URU}-a-tu$_2$ li-ir-a šu-bal-lu-u$_2$ a-

BiLSTM: a-na DA ri$_2$ a-tam-li-ir-a-šu pal-lu-u$_2$ a

l.35: 𒇷xx𒌨𒋻𒆕𒀀𒈾𒈗𒌑𒋾𒅀

transl.: li-x-x lik-tar-rab a-na LUGAL-u$_2$-ti-ia

HMM: li-x x ur-tar-rap-a-na LUGAL-u$_2$-ti-ia

MEMM: li-x x EŠ tar-rab-a-na LUGAL-u$_2$-ti-ia

BiLSTM: li-x x ur tar-rab a-na LUGAL-u$_2$-ti-ia

A Hellenistic Royal Inscription of Antiochus I, ca. 281-261, found in Borsippa (Q00417)

l.1: [illegible]

transl.: ^{m}an-ti-ʾu-ku-us LUGAL GAL-u_2

HMM: {m}-{d}-ti aʾ-ku-us-LUGAL GAL-u_2

MEMM: {m}-{d}-ti-ʾi-ku-us LUGAL GAL-u_2

BiLSTM: {m}-{d}-ti-ʾi-ku-u_2 LUGAL GAL-u_2

l.2: [illegible]

transl.: LUGAL dan-nu LUGAL $ŠAR_2$ LUGAL E.KI LUGAL KUR.KUR

HMM: LUGAL dan-nu LUGAL $ŠAR_2$ LUGAL e-{KI} LUGAL {KUR}-{KUR}-

MEMM: LUGAL dan-nu LUGAL KIŠ LUGAL e ki-LUGAL KUR.KUR

BiLSTM: LUGAL dan-nu LUGAL $ŠAR_2$ lugal e-{KI} LUGAL KUR.KUR

l.3: [illegible]

transl.: za-ni-in e_2-sag-il_2 u_3 e_2-zi-da

HMM: ṣa-ni in-e_2-sag-il_2 u_3 E_2.zi-da-

MEMM: za-ni-in-e_2-sag-il_2 u_3 E_2 zi-da

BiLSTM: za-ni-in-e_2-sag-il_2 u_3 e_2-zi-da

l.4: [illegible]

transl.: IBILA SAG.KAL ša ^{m}si-lu-uk-ku LUGAL

HMM: DUMU uš-šak-reb ša {m}-si-lu-uk-ku-LUGAL

MEMM: DUMU uš-SAG.kal-ša {m}-si-lu-uk-ku-LUGAL

BiLSTM: DUMU uš-šak-dan-ša {m}-si-lu-uk-ku LUGAL

l.5: [illegible]

transl.: LU_2.ma-ak-ka-du-na-a-a LUGAL E.KI

HMM: {LU_2}-ma-ak-ka-du-na-a-a-LUGAL e-ki

MEMM: {LU_2}-ma-aq-ka-du-na-a-a LUGAL e-ki-

BiLSTM: {LU_2}-ma-ak-ka-du-na-a-a LUGAL-e-{KI}

l.6: [illegible]

transl.: a-na-ku i-nu-ma a-na e-pe_2-$eš_{15}$

HMM: a-na ku-i-nu ma a-na e-bi-iš-

MEMM: a-na ku i-nu-ma a-na e-bi-iš-

BiLSTM: a-na-ku i-nu-ma a-na e-bi-iš

l.7: [illegible]

transl.: e_2-sag-il_2 u_3 e_2-zi-da

HMM: e_2-sag-il_2 u_3 E_2.zi-da-

MEMM: e_2-sag-il_2 u_3 E_2 zi-da

BiLSTM: e_2-sag-il_2 u_3 e_2-zi-da

l.8: [illegible]

transl.: lib_3-bi ub-lam-ma SIG_4.ḪI.A

HMM: lib_3-bi-ub-lam-ma SIG_4 hi-a-

MEMM: lib_3-bi $iš_8$-tar_2 ma ŠIM.HI.A

BiLSTM: lib₃-bi ub-lam-ma SIG₄.HI.A

l.9: 𒂍𒊕𒅍𒅇𒂍𒍣𒁕

transl.: e₂-sag-il₂ u₃ e₂-zi-da

HMM: e₂-sag-il₂ u₃ E₂.zi-da-

MEMM: e₂-sag-il₂ u₃ E₂ zi-da

BiLSTM: e₂-sag-il₂ u₃ e₂-zi-da

l.10: 𒀸𒆳𒄩𒀜𒁴𒀸𒋗𒈫𒅀𒂖𒇷𒋾

transl.: ina KUR.ḫa-at-tim ina ŠU-MIN-ia₂ el-le-ti

HMM: ina {KUR}-ha-at-tim ina šu-2 el-li-ti

MEMM: ina {KUR}-ha-at-tim ina ŠU.II ia₂ el-li-ti

BiLSTM: ina {KUR}-ha-at-tim ina ŠU.2.ia₂ el-li-ti

l.11: 𒄿𒈾𒉌𒄑𒊒𒍑𒋾𒀠𒁉𒅔𒈠

transl.: i-na I₃.GIŠ ru-uš-ti al-bi-in-ma

HMM: i-na ni {GIŠ}-ru-uš-ti al-bi-in-ma

MEMM: i-na ni {GIŠ}-ru-uš ti al-bi-in-ma

BiLSTM: i-na I₃.GIŠ ru-uš-ti al-bi-in-ma

l.12: 𒀀𒈾𒈾𒁲𒂊𒍑𒋗𒊭𒂍𒊕𒅍

transl.: a-na na-de-e uš-šu ša e₂-sag-il₂

HMM: a-na na di e-uš-šu-ša e₂-sag-il₂

MEMM: a-na na-de-e uš-šu ša E₂-sag-il₂

BiLSTM: a-na na-de-e-uš-šu ša e₂-sag-il₂

l.13: 𒅇𒂍𒍣𒁕𒌒𒉈𒅋𒀸𒌚𒊺𒌓𒎙𒄰

transl.: u₃ e₂-zi-da ub-bi-il ina ITI.ŠE UD.20.KAM

HMM: u₃ E₂.zi-da-ub bi-il-ina {ITI}-še-tu₂ u u kam-

MEMM: u₃ E₂ zi-da up-bi-il-ina HUL-še-tu₂ MAN KAL

BiLSTM: u₃ e₂-zi-da-ub-bi-il ina {ITI}-ŠE UD.20.KAM

l.14: 𒈬𒐏𒐈𒄰𒍑𒋗𒊭𒂍𒍣𒁕

transl.: MU.43.KAM uš-šu ša e₂-zi-da

HMM: mu-40 3 kam-uš-šu-ša E₂.zi-da-

MEMM: mu-suk-kan-kam-uš-šu ša E₂-zi-da

BiLSTM: mu 40 3.KAM uš-šu ša e₂-zi-da

l.15: 𒂍𒆠𒄿𒉌𒂍𒀭𒀝𒃻𒆠𒊑𒁇𒆠

transl.: E₂ ki-i-ni E₂ ^{d}AG ša₂ qe₂-reb BAR-SIP₃.KI

HMM: E₂.{KI} i-ni E₂.{d}-ak-ša₂ {KI} reb BAR ṣip {KI}

MEMM: e₂-ki-i ni-tum {d}-AG ša₂ qe₂-reb sipa {KI}

BiLSTM: bit-ki-i-ne₂-E₂ {d}-AG ša₂ qe₂-reb BAR {KI}

l.16: 𒀜𒁲𒂊𒍑𒅆𒋗𒀭𒀝𒌉𒍑𒍢𒄿𒊑

transl.: ad-de-e uš-ši-šu ^{d}AG IBILA ṣi-i-ri

HMM: at-di e-uš-ši-šu-{d}-ak-DUMU uš-ṣe-i-ri-

MEMM: ad-de-e uš-IGI.šu {d}-AG DUMU uš-ṣi-i-ri

BiLSTM: ad-de-e uš-ši-šu {d}-AG IBILA ṣi-i-ri

l.17: [illegible]

transl.: IGI.GAL$_2$.LA DINGIR.MEŠ muš-tar-ḫu

HMM: ši-ik-la DINGIR.MEŠ ṣir tar-{MUŠEN}

MEMM: ši-ik-la-DINGIR.MEŠ PAP tar-hu-

BiLSTM: ši-ik-LA DINGIR.MEŠ muš-tar-hu

l.18: [illegible]

transl.: ša a-na ta-na-da-a-ti

HMM: ša a-na ta-na-da-a-ti

MEMM: ša a-na ta-na-da-a-ti

BiLSTM: ša a-na ta-na-da-a-ti

l.19: [illegible]

transl.: šit-ku-nu IBILA reš-tu-u$_2$

HMM: šit-ku-nu DUMU uš-šak-tu u$_2$-

MEMM: SANGA-ku-nu ENSI$_2$ uš-riš tu u$_2$-

BiLSTM: šit-ku-nu IBILA reš-tu-u$_2$

l.20: [illegible]

transl.: ša ^{d}AMAR.UTU i-lit-ti ^{d}e$_4$-ru$_6$-u$_2$-a

HMM: ša {d}-AMAR.tu$_2$ i-lit-ti {d}-a-EDIN u$_2$-a

MEMM: ša {d}-AMAR.UTU i-mat-ti {d}-a-nun-u$_2$-a-

BiLSTM: ša {d}-AMAR.UTU i-lit-ti {d}-A.EDIN u$_2$-a-

l.21: [illegible]

transl.: šar-rat pa-ti-qat$_2$ nab-ni-ti

HMM: šar-rat pa-ti šu-2 nap-ni ti

MEMM: šar-rat LIMMU$_2$-ti šu 2 nab-ni-ti

BiLSTM: šar rat pa-ti-šu-2 nab-ni-ti

l.22: [illegible]

transl.: ḫa-diš nap-li-is-ma

HMM: ha-{m}-nap-li-{GIŠ}-ma

MEMM: ha-{m}-til-li-{GIŠ}-ma

BiLSTM: ha-diš nap-li-iz-ma

l.23: [illegible]

transl.: i-na qi$_2$-bi-ti-ka ṣi-ir-ti

HMM: i-na qi$_2$-bi-ti ka-ṣi-ir-ti

MEMM: i-na ki-bi ti ka-ṣi-ir ti

BiLSTM: i-na qi$_2$-bi-ti-ka ṣi-ir-ti

l.24: [illegible]

transl.: ša la in-nen-nu-u$_2$ qi$_2$-bit-su

HMM: ša la in-šal-nu u$_2$-{KI} E$_2$.su-

MEMM: ša la-in nin nu-u$_2$-ki-bit-su-

BiLSTM: ša la in-nen-nu-u$_2$ qi$_2$-bit-su

l.25: [illegible]

transl.: šu-um-qu-ut ma-a-ti a-a-bi-ia$_2$

HMM: šu-um-qu-tu$_2$ ma-a-ti a-a-bi-

MEMM: šu-um-qu-ut-ma a-ti a-a-bi-5

BiLSTM: šu-um-qu-tam-ma-a-ti a-a-bi-ia$_2$

l.26: [illegible]

transl.: ka-ša$_2$-du er-ni-it-ti-ia$_2$

HMM: ka-ša$_2$ du-ir-ni it-ti

MEMM: ka-ša$_2$-du ir-ni-it-ti ia$_2$

BiLSTM: ša$_2$-du-er-ni it-ti-ia$_2$

l.27: [illegible]

transl.: UGU na-ki-ri u$_2$-šu-uz-zu i-na li-i-ti

HMM: u ka-na {KI} ri-u$_2$-šu-us-zu i-na li-i-ti

MEMM: UGU na-ki-ri u$_2$-šu-us zu-i-na li-i-ti

BiLSTM: UGU na-ki-ri u$_2$-šu-uz-zu i-na li-i-ti

l.28: [illegible]

transl.: LUGAL-u$_2$-tu mi-ša$_2$-ri pa-le-e

HMM: LUGAL-u$_2$-tu mi-ša$_2$ ri-pa-li-e-

MEMM: LUGAL-u$_2$-tu mi-ša$_2$ ri pa-le-e-

BiLSTM: LUGAL-u$_2$-tu mi-ša$_2$-ri pa-li e

l.29: [illegible]

transl.: bu-a-ri MU.AN.NA.MEŠ ṭu-ub lib$_3$-bi

HMM: bu-a-ri-MU.AN.NA.MEŠ ṭu-ub lib$_3$-bi-

MEMM: pu-a-ri-mu {d}-na-MEŠ ṭu-up-lib$_3$-bi

BiLSTM: bu a-ri MU.AN.NA.MEŠ ṭu-ub lib$_3$-bi

l.30: [illegible]

transl.: še-be$_2$-e lit-tu-tu lu ši-ri-ik-ti

HMM: še-be$_2$-e lit-tu-tu lu-ši-ri-ik-ti

MEMM: še-be$_2$-e-tel-tu tu lu ši-ri-ik ti

BiLSTM: še-be$_2$-e lit-tu-tu lu-ši-re-iq-ti

l.31: [illegible]

transl.: LUGAL-u$_2$-ti ša$_2$ ^{m}an-ti-ʾu-ku-us

HMM: LUGAL-u$_2$-ti ša$_2$ {m}-{d}-ti aʾ-ku-us-

MEMM: LUGAL-u$_2$-ti ša$_2$ {m}-{d}-ti-ʾi-ku-us

BiLSTM: LUGAL-u$_2$-ti ša$_2$ {m}-DINGIR-ti-ʾi-ku-uṣ

l.32: [illegible]

transl.: u$_3$ si-lu-uk-ku LUGAL DUMU-šu$_2$

HMM: u$_3$ si-lu-uk-ku-LUGAL DUMU šu$_2$

MEMM: u$_3$ si-lu-uk-ku-LUGAL DUMU šu$_2$

BiLSTM: u$_3$ si-lu-uk-ku LUGAL DUMU-šu$_2$

l.33: [illegible]

transl.: a-na da-ra-a-ti DUMU ru-be$_2$-e

HMM: a-na da-ra-a-ti DUMU ru-bi-e-

MEMM: a-na da-ra-a-ti DUMU ru-be$_2$-e

BiLSTM: a-na da-ra-a-ti DUMU ru-be$_2$-e

l.34: [illegible]

transl.: ^{d}AG IBILA e$_2$-sag-il$_2$

HMM: {d}-ak-DUMU uš-E$_2$.šak-il$_2$

MEMM: {d}-AG DUMU uš-e$_2$-sag-il$_2$

BiLSTM: {d}-AG IBILA e$_2$-sag-il$_2$

l.35: [illegible]

transl.: bu-kur$_2$ ^{d}asar-ri reš-tu-u$_2$

HMM: bu-PAP.{d}-asal-ri-reš-tu-u$_2$

MEMM: bu-tum {d}-U.ri-riš tu u$_2$-

BiLSTM: bu-sun$_2$ {d}-asar-ri reš-tu-u$_2$

l.36: [illegible]

transl.: i-lit-ti ^{d}e$_4$-ru$_6$-u$_2$-a šar-rat

HMM: i-lit-ti {d}-a-EDIN u$_2$-a šar-rat

MEMM: i-mat-ti {d}-a-nun-u$_2$-a-šar-rat

BiLSTM: i-lit-ti {d}-A.EDIN u$_2$-a-šar-rat

l.37: [illegible]

transl.: a-na e$_2$-zi-da E$_2$ ki-i-ni

HMM: a-na E$_2$.zi-da-E$_2$.{KI} i-ni

MEMM: a-na E$_2$ zi-da e$_2$-ki-i ni-

BiLSTM: a-na e$_2$-zi-da E$_2$ ki-i ni-

l.38: [illegible]

transl.: E$_2$ da-nu-ti-ka šu-bat ṭu-<ub> lib$_3$-bi-ka

HMM: E$_2$ {d}-a-nu-ti ka-šu-be-ṭu-ub lib$_3$-bi-ka

MEMM: E$_2$ {d}-a-nu-ti ka-šu-bat ṭu-up-lib$_3$-bi ka-

BiLSTM: bit {d}-a-nu-ti-ka šu-bat ṭu-ub lib$_3$-bi-ka

l.39: [illegible]

transl.: i-na ḫi-da-a-tu$_2$ u$_3$ ri-ša$_2$-a-tu$_2$

HMM: i-na hi-ṭa-a-tu$_2$ u$_3$ ri-ša$_2$-a-tu$_2$

MEMM: i-na hi-da-a-tu$_2$ u$_3$ ri ša$_2$ a-tu$_2$

BiLSTM: i-na hi-ṭa-a-tu$_2$ u$_3$ ri ša$_2$-a-tu$_2$

l.40: [illegible]

transl.: i-na e-re-bi-ka i-na qi$_2$-bi-ti-ka

HMM: i-na e-ri-bi-ka i-na qi$_2$-bi-ti ka-

MEMM: i-na e-ri-bi ka-i-na ki-bi ti ka-

BiLSTM: i-na e-ri-bi-ka i-na qi$_2$-bi-ti-ka

l.41: [illegible]

transl.: kit-ti ša la uš-tam-sa-ku li-ri-ku u$_4$-mi-ia$_2$

HMM: kit-ti ša la uš-tu$_2$ sa-ku-li-ri-ku-tu$_2$ mi-

MEMM: kit-ti ša la-uš-tu$_2$ sa-ku li-ri ku-tu$_2$ mi-5

BiLSTM: kit-ti ša la uš-tu$_2$ sa-ku li-tal-ku-ut mi-ia$_2$

l.42: [illegible]

transl.: li-mi-da MU.AN.NA-ti-ia$_2$

HMM: li-mi-da-mu-{d}-na ti

MEMM: li-mi-da-mu {d}-na-ti-ia$_2$

BiLSTM: li-mi-da-mu an-na-ti-ia$_2$

l.43: [illegible]

transl.: li-kun GIŠ.GU.ZA-u$_2$-a li-il-bi-ir

HMM: li-kun {GIŠ}-gu-ṣa-u$_2$-a-li il-bi-ir-

MEMM: li-kun-{GIŠ}-GU.ZA-u$_2$-a-li-il-bi-ir-

BiLSTM: li-kun {GIŠ}-GU.ZA-u$_2$-a li il-bi-er

l.44: [illegible]

transl.: pa-lu-u$_2$-a i-na GIŠ.DA-ka ṣi-i-ri

HMM: pa-lu-u$_2$-a i-na {GIŠ}-da-ka-ṣe-i-ri-

MEMM: pa-lu-u$_2$ a i-na {GIŠ}-da-ka-ṣi-i-ri

BiLSTM: pa-lu-u$_2$-a i-na {GIŠ}-da-ka ṣi i-ri

l.45: [illegible]

transl.: mu-kin pal-lu-uk-ku? AN-e u KI-tim

HMM: mu-kin bal-lu-uk-ku-{d}-e-u {KI} tim

MEMM: mu-kin bal-lu-uk-ku-AN-e mut-tim

BiLSTM: mu-kin pal-lu-uk-ku AN-e u KI-tim

l.46: [illegible]

transl.: i-na pi-i-ka el-li liš-tak-ka-nu

HMM: i-na pi-i-ka-el-li-liš tak-ka-nu

MEMM: i-na pi-i-ka-el-li-liš tak-ka-nu

BiLSTM: i-na pi-i-ka el-li liš tak-ka-nu-

l.47: [illegible]

transl.: du-un-qi$_2$-ia$_2$ KUR.KUR.MEŠ TA ṣi-it ^{d}UTU-ši

HMM: du-un {KI} lat KUR.MEŠ TA ṣi-it {d}-UTU-ši

MEMM: du-un ki-5 KUR.KUR.MEŠ ta-ṣi-it {d}-UTU-ši

BiLSTM: du-un-ki-ia$_2$ KUR.KUR.MEŠ ta-ṣi-it {d}-UTU-ši

l.48: [illegible]

transl.: a-di e-re-eb ^{d}UTU-ši lik-šu-du

HMM: a-di e-ri-ip-{d}-tu$_2$ ši-ur-šu-du-

MEMM: a-di e-ri-ep-{d}-UTU-ši ur-šu-du

BiLSTM: a-di e-ri-ib {d}-UTU-ši šu-du-

l.49: [illegible]

transl.: ŠU-MIN-a-a man-da-at-ti-ši-nu lu-us-ni-iq-ma

HMM: šu-2 a-a man-da-at-ti ši-nu lu-us-ni iq-ma

MEMM: šu-2 a-a MAN da-at-ti-ši-nu lu-uz-ni-ik-ma

BiLSTM: šu 2.a-a man-da-at-ti lem-nu-lu-uṣ-ni-iq-ma

l.50: [illegible]

transl.: a-na šuk-lu-lu e$_2$-sag-il$_2$

HMM: a-na šuk-lu-lu-e$_2$-sag-il$_2$

MEMM: a-na šuk-lu-lu-e$_2$-sag-il$_2$

BiLSTM: a-na šuk-lu-lu e$_2$-sag-il$_2$

l.51: [illegible]

transl.: u$_3$ e$_2$-zi-da lu-bi-il ^{d}AG

HMM: u$_3$ E$_2$.zi-da-lu-bi-il-{d}-ak-

MEMM: u$_3$ E$_2$ zi-da lu-bi-il-{d}-AG

BiLSTM: u$_3$ e$_2$-zi-da lu-bi-il an AG

l.52: [illegible]

transl.: IBILA SAG.KAL a-na e$_{2}$-zi-da

HMM: DUMU uš-šak-rib a-na E$_{2}$.zi-da-

MEMM: DUMU uš-SAG.lab-a-na E$_{2}$ zi-da

BiLSTM: DUMU SAG.kal-a-na e$_{2}$-zi-da

l.53: [illegible]

transl.: E$_{2}$ ki-i-ni i-na e-re-bi-ka

HMM: E$_{2}$.{KI} i-ni i-na e-ri-bi-ka-

MEMM: e$_{2}$-ki-i ni-i-na e-ri-bi ka-

BiLSTM: E$_{2}$ ki-i-ni i-na e-ri-bi-ka

l.54: [illegible]

transl.: SIG$_{5}$-ti$_{3}$ ^{m}an-ti-ʾu-ku-us LUGAL KUR.KUR

HMM: ši-ERIM.tim {m}-{d}-ti aʾ-ku-us-LUGAL {KUR}-{KUR}-

MEMM: ši ERIM.tim {m}-{d}-ti-ʾi-ku-us LUGAL KUR.KUR

BiLSTM: SIG$_{5}$-tim {m}-DINGIR ti-iʾ-ku-us LUGAL KUR.KUR

l.55: [illegible]

transl.: ^{m}si-lu-uk-ku LUGAL DUMU-šu$_{2}$

HMM: {m}-si-lu-uk-ku-LUGAL DUMU šu$_{2}$

MEMM: {m}-si-lu-uk-ku-LUGAL DUMU šu$_{2}$

BiLSTM: {m}-si-lu-uk-ku LUGAL DUMU-šu$_{2}$

l.56: [illegible]

transl.: ^{f}as-ta-ar-ta-ni-ik-ku

HMM: šal-as-ta-ar-ta-ni-ik-ku-

MEMM: SIG$_{3}$-aṣ-ta-ar-ta-ni ik-ku-

BiLSTM: šal-as-ta-ar-ta-ni iq ku

l.57: [illegible]

transl.: ḫi-rat-su šar-ra-at

HMM: hi-rat su-šar-ra-at-

MEMM: hi-im su šar-ra-ad-

BiLSTM: hi-rat-su šar-ra-ad-

l.58: [illegible]

transl.: da-mi-iq-ti-šu$_{2}$-nu

HMM: da-mi-iq-ti-šu$_{2}$-nu

MEMM: da mi-ik-ti-šu$_{2}$-nu

BiLSTM: da-mi-iq-ti-šu$_{2}$-nu

l.59: [illegible]

transl.: li-iš-ša$_{2}$-kin i-na pi-i-ka

HMM: li-iš-ša$_{2}$ kin i-na pi-i-ka

MEMM: li-iš-ša$_{2}$-qi-i-na pi-i-ka-

BiLSTM: li-iš-ša$_{2}$-kin i-na pi-i-ka
